# Health inequalities among migrant and native-born populations in Greece in times of crisis: the MIGHEAL study.

**DOI:** 10.1093/eurpub/cky225

**Published:** 2018-11-21

**Authors:** Theoni Stathopoulou, Per Stornes, Aliki Mouriki, Anastasia Kostaki, Jennifer Cavounidis, Lydia Avrami, Courtney L McNamara, Carolin Rapp, Terje A Eikemo

**Affiliations:** 1 National Centre for Social Research, Athens, Greece; 2 Centre for Global Health Inequalities Research (CHAIN), Department of Sociology and Political Science, Norwegian University of Science and Technology (NTNU), Trondheim, Norway; 3 Department of Statistics, Athens University of Economics and Business, Athens, Greece; 4 Department of Economics, Athens University of Economics and Business, Athens, Greece; 5 Department of Political Science, University of Copenhagen, Copenhagen, Denmark

## Abstract

This article presents the MIGHEAL study, which was developed in parallel with the European Social Survey (ESS) Round 7 (2014). Conducted in Greece in 2016 by the National Centre for Social Research, the study was specifically designed to further our understanding of how health varies by social status, focusing particularly on migrant status. In the current article, we report results on health status (non-communicable diseases, self-reported health and depressive symptoms) and health determinants (risky health behaviours, social determinants and access to health care) in Greece, among migrants and native-born. Estimates for the Greek overall population are compared with the European ones (using the ESS 2014 data) and discussed with reference to the ongoing economic and social crisis in Greece. The study provides evidence of social inequalities in health, complementing the pan-European documentation, and supports prior research, which has identified negative health consequences of the crisis.

## Introduction

In times of economic polarization, historically high unemployment levels and growing social deprivation, it is of particular importance to collect data at the population level that can help us understand the impact of these factors on health. Because economic recession typically hits hardest among the most disadvantaged groups, it is likely to lead not only to the deterioration of overall population health but also to increased inequality in health outcomes and in access to healthcare services.^1^

To understand how the Greek crisis has impacted health and health inequalities in Greece, we need to address how the crisis may have affected key health determinants in the country. The first group of such determinants is ‘risky health behaviours’. A study comparing three waves of the Greek cross-sectional household ‘Hellas Health’ surveys showed that fruit and vegetable consumption dramatically declined, during the economic crisis in Greece.^2^ While trends for smoking and physical activity were positive among all socio-economic groups in the period, social disparities in fruit and vegetable consumption, physical activity and smoking were observed in all three surveys. These results suggest that the Greek crisis has had both positive and negative effects on health behaviours.

The second group of determinants that may have been affected by the crisis comprises wider ‘social determinants’, such as working conditions, financial strain and quality of housing. The crisis has had the greatest impact on the disadvantaged, on those working under poor occupational conditions, and on low-income households, as these are more vulnerable to income reduction and are more likely to suffer the labour market effects of an economic crisis.^3^

The third group of health determinants relates to ‘healthcare’. The effects of the economic crisis and accompanying austerity measures imposed on the health sector in Greece have been visible in terms of spending cuts for medical goods and services (up to 25%), reductions in the number of available hospital beds (from 35 000 to 33 000), the merging of public clinics and an increase in unemployment rates among young physicians.^4^ Moreover, admissions to and utilization of public health services increased by 30% between 2011 and 2013 as a result of people’s inability to pay for private healthcare.^5^^,^^6^ Additionally, over 6% of the general population reported some unmet medical care need for financial reasons during 2011 and 2012 compared with 4% in 2008. The proportion reached 11% among people in the lowest income quintiles in 2012, up from 7% in 2008.^7^ In addition, between 2010 and 2011, funding for mental health care was reduced by 20%, and by a further 55% between 2011 and 2012. While this diminished the capacity of mental health services to meet medical needs, an increase of 120% in the use of such services was recorded.^8^ A time-series analysis for the years 2004 through 2011 using the EU-SILC database reported that the adverse economic environment has significantly affected unmet health needs in Greece.^9^ The effects of the crisis on healthcare provision are reflected in the percentage of Greeks who have sought healthcare through non-governmental organizations during the crisis. According to a report by Doctors of the World, in 2012, Greek nationals constituted approximately half (49.3%) of the patients treated in its four clinics established in Greece.^10^

There is currently a lack of knowledge as to how exactly these determinants are associated with health status in Greece in the aftermath of the crisis. Moreover, these health determinants are likely to have affected migrants and natives differently. For example, migrants are often reported to be more susceptible to multiple discrimination when it comes to access to and quality of healthcare.^11^^,^^12^ For undocumented migrants, entitlement to healthcare varies substantially across European countries and is hindered by formal and informal barriers.^12^ At a European level, Solé-Auró and Crimmins have shown that the health status of migrants varies considerably by country of origin and gender.^13^ Evidence from both the USA and Europe suggests that migrants are sometimes healthier than might be expected^14^, though research in Europe has found mixed patterns of migrant health depending on country of origin.^15^ Still, we lack evidence as to how migrants and natives in Greece have been affected differently by the crisis both domestically and in comparison with other European countries, thus pointing to the necessity of the MIGHEAL study.

### MIGHEAL and the European Social Survey

The MIGHEAL survey was designed and conducted in line with the specifications of Round 7 of the European Social Survey (ESS), which included a health module and in which Greece did not participate. The coding of variables and the adaptation of specific measures (e.g. alcohol consumption) were done in accordance with the standards of the ESS. The MIGHEAL dataset can therefore be used for cross-national comparisons with Europe after appropriate data harmonization.

Twenty-one countries participated in the ESS Round 7 (2014), with national representative sample sizes ranging from 1224 to 3045, and the total reaching 40 185 respondents. Likewise, MIGHEAL is a representative population sample of Greece, with a sample size of 1332 respondents, also designed to be representative of the migrant population, undocumented or not. Thus, the MIGHEAL sample consists of 827 natives and 505 migrants. To obtain population size estimates, the Greek population was weighted up to around 1200 cases, and the migrant population was weighted down to around 100 cases using the population size weight in the MIGHEAL dataset.

## Methodology

### The MIGHEAL sampling frame

The MIGHEAL sample population is divided into two groups—migrants and non-migrants—based on the respondents’ country of birth. Respondents who are foreign-born (first-generation migrants) or native-born but with at least one foreign-born parent (second-generation migrants) are classified as migrants. Respondents born in one of the EU member countries or in developed countries outside the EU are not classified as migrants. The World Bank’s definition of high-income countries as those with a GDP per capita of US$37 755 or more was adopted to designate countries as ‘developed’ or not.

In the MIGHEAL dataset, the native population comprises individuals aged 15 or over living in private households. Enumeration preceded fieldwork in order to establish a probabilistic sample for the survey. The primary sampling unit (PSU) selection for enumeration was derived by a sampling framework incorporating all possible areas/settlements and randomly selecting PSUs (according to the 2011 Census). After enumeration, addresses were randomly selected and included in the sample. Random selection of respondents (KISH grid) occurs when an eligible household is identified. Selected households and selected respondents were not replaced, and at least four visits/contacts were made to each selected household before attributing it a final code.

The migrant population comprises individuals aged 15 or over living in private households and having sufficient knowledge of the Greek language. The PSU selection for enumeration was derived by a sampling framework incorporating all possible areas/settlements and randomly selecting PSUs, by approaching the areas with higher density of target groups (according to the 2011 Census).

In addition, focused enumeration was used for the migrant population. Sample selection was based on geographical stratification on an NUTS2 level, proportionally to the population data of urban areas and census enumeration, in order to establish a probability sample for the survey. More specifically, 128 surface units were targeted on the basis of migrant population density in each area. These surface units were distributed proportionally to the geographical strata. In each surface area, households were enumerated starting from the northeast corner of the surface area and moving clockwise. Then, 18 households were randomly selected in each surface unit and included in the sample.

To ensure the comparability of MIGHEAL with ESS data, the migrant population sample was drawn using a multi-stage stratified sampling technique. More specifically, the 13 administrative regions of the country were considered as strata and the sample size of each region was proportional to the size of the immigrant population in each region according to the 2011 population census. In total, 80 sampling sites from the 13 strata were selected to identify individuals who belonged to the survey population of migrants. For the native population, 80 sampling sites were also randomly selected, close to those selected for the migrant population, to ensure comparability between the two population groups (matched geographic sample). Another 48 sampling points from the urban areas of the country were also randomly selected so that the resulting final sample of the native population approximated the population distribution of the urban population of the country by region according to the 2011 population census (boost sample).

Data collection mode was paper and pencil interviews. Fieldwork was conducted from 19 May to 28 July 2016 across Greece, and the response rate was 50%.

### Fieldwork and questionnaire design

The MIGHEAL survey questionnaire was identical for both migrants and non-migrants. The questionnaire was mostly based on the core ESS questionnaire, the ESS Round 7 Health Module^16^ and the ESS Round 7 Immigrant Module,^17^ with some additions from the Hellenic Statistical Authority’s Health Survey^18^ and the Canadian Longitudinal Survey for migrants (table 1).^19^ Adaptation of specific measures (i.e. alcohol consumption) for Greece was performed after consultation with the ESS team.

**Table 1:** Core MIGHEAL variables

Variables	Source
Attitudes towards immigration	Core ESS questionnaire
Discrimination	Core ESS questionnaire
Happiness and well-being	Core ESS questionnaire
Household income	Core ESS questionnaire
Interpersonal trust	Core ESS questionnaire
Occupational status	Core ESS questionnaire
Religion	Core ESS questionnaire
Satisfaction with health system	Core ESS questionnaire
Self-reported general health	Core ESS questionnaire
Qualification for immigration	ESS Round 7 Immigrant Module
Alternative treatments	ESS Round 7 Health Module
Depression	ESS Round 7 Health Module
Family background (conflict while growing up, economic hardship)	ESS Round 7 Health Module
Health use (GP, medical specialists)	ESS Round 7 Health Module
Lifestyle (sports, smoking, alcohol)	ESS Round 7 Health Module
Limiting long-standing illness	ESS Round 7 Health Module
Physical working conditions	ESS Round 7 Health Module
Self-reported conditions	ESS Round 7 Health Module
Unmet need (reasons for not getting medical consultation or treatment)	ESS Round 7 Health Module
Unpaid care	ESS Round 7 Health Module
Vision/hearing problems	Greek National Health Survey 2014
Use of visual/hearing aids	Greek National Health Survey 2014
Barriers to access (language, beliefs)	Longitudinal Survey of Immigrants to Canada 2005

The pilot survey of the questionnaire was carried out from 22 April to 24 April 2016 with a sample of 10 migrants and 10 non-migrants residing in Attica. The final questionnaire was revised in line with the comments obtained from the pilot survey.

### Cognitive testing

Qualitative methods were applied to explore whether the proposed survey questions were comprehensible, acceptable, unobtrusive, valid and comparable across ethnic groups, religions and age groups. Cognitive testing was performed through 15 interviews with 5 non-migrants and 10 migrants of the following nationalities: Albanian (6 individuals), Pakistani (2), Georgian (1) and Ukrainian (1). The cognitive interviews lasted one hour and were recorded before being transcribed and analyzed.

### The MIGHEAL data

Respondents were divided into three groups based on citizenship: (i) Greek citizens, (11) Albanian citizens and (iii) other country citizens, from former socialist Central and Eastern European countries, as well as from Asia, Africa and the Middle East.

The migrant population in the MIGHEAL survey had a mean length of stay in Greece of 14–16 years and a lower average age than the non-migrant population. There were no Albanian or other country men over the age of 64 in the sample and very few migrant women over the same age. As age is inherently associated with many health outcomes, for the purpose of analysis the sample was limited to respondents between 20 and 64 years of age to achieve adequate comparisons. The process resulted in a total raw sample of 1006 respondents (see table 2 for distribution).

**Table 2:** Population groups in MIGHEAL (capped)

Citizenship	Male	Female	Total
Greek	256	311	567
Albanian	158	122	280
Other country	107	52	159
Total	521	485	1006

Men originating from other countries in the MIGHEAL sample are from 18 different countries, with Pakistan accounting for the largest group, followed by Bangladesh and Egypt. Women originate from 19 different countries, the largest group coming from Georgia and the second largest from Ukraine.

An overview of the main sample characteristics is provided in table 3.

**Table 3:** MIGHEAL sample characteristics

Measure	Greek citizens	Albanian citizens	Other country citizens
Education	Secondary/	Lower secondary	Upper secondary
	Tertiary		
Income	3rd decile	2nd decile	3rd decile
	(454–681 E)	(227–454 E)	(454–681 E)
Paid work	>50%	60%	70%
Unemployed	20%	20%	20%
Mean age	42 M/F	41 M/36 F	38 M/42 F
Coping with household income:	40%	20%	25%
Mean age of arrival		20 years	25 years
Mean length of stay		16 years	14 years
Mean year of arrival		2000	2002
Main countries of origin			Pakistan/Bangladesh (M)
			Georgia (F)

## Analysis

Our analysis focuses on the comparison between Greece, based on the MIGHEAL data, and the 21 counties from the 2014 ESS. The analysis hinges on two approaches: at a first instance, we calculate prevalence rates of non-communicable diseases (NCDs), self-reported health, healthcare usage, health behaviours and social determinants. These rates are stratified by gender, in addition to being age-standardized according to the European Standard Population 2013. For the ESS countries, the post-stratification weight is applied, while for the MIGHEAL data, the population size weight is applied. At a second instance, we apply logistic regression analysis to test the relationship between the citizenship of individuals living in Greece and indicators related to general health, disease, risk behaviour and determinants of health. For all analyses, the measures are treated as dummy variables, except for units of alcohol where mean units are specified. For units of alcohol on weekdays and weekends, a regular regression analysis is used in order to predict the number of units based on citizenship. We are not referring to confidence intervals in our presentation of the point estimates below, but prevalences and odds ratios are reported with 95% confidence intervals in tables and figures.

## Results

In this section, we first present the prevalence of self-reported health status, self-reported depressive symptoms, health care use and unmet needs in both the MIGHEAL and the ESS data. All percentages are age-standardized. Tables 5–9 present our findings. Apart from the simple prevalence rates, we present logistic regression results in table 11, which test the occurrence of health issues and health behaviour based on citizenship. Table 4 gives an overview of the variables and their measurements.

**Table 4:** Concepts and measurement used

Concepts	Description of measurement used
Physical activity	Number of days on which respondents walked quickly, did sports or other physical activity for 30 min or longer in the last 7 days
Smoking	Self-classifying as a daily smoker, occasional smoker, former smoker or non-smoker (cigarettes and rolled tobacco, excluding pipes, cigars and electronic cigarettes)
	Number of cigarettes smoked on a typical day
Alcohol consumption	Frequency of alcohol consumption in the last 12 months
	Number of drinks (as displayed on showcard) consumed the last time drinking alcohol on a Monday, Tuesday, Wednesday or Thursday
	Number of drinks (as displayed on showcard) consumed the last time drinking alcohol on a Friday, Saturday or Sunday
	Frequency of binge drinking in the last 12 months
BMI	Height without shoes; weight without shoes
Health care utilization	Discussed health with a general practitioner during the past 12 months
	Discussed health with a medical specialist during the past 12 months
	Unable to get a medical consultation or the treatment needed during the past 12 months
	Reasons for being unable to get a medical consultation or the treatment needed during the past 12 months
	Alternative treatments used in the last 12 months (12 types of treatment on showcard)
Provision of unpaid care	Looking after or giving help to family members, friends, neighbours or others because of long-term physical or mental ill health or disability, or problems related to old age, not counting paid employment
	Hours per week spent providing unpaid care
Dimensions of mental well-being	Felt depressed; everything was an effort; sleep was restless; happy; lonely; enjoyed life; sad; could not get going; during the past week (8 separate items)
Self-reported conditions	Health problems (on showcard) experienced in the last 12 months: heart or circulation problem; high blood pressure; breathing problems; allergies; back or neck pain; muscular or joint pain in hand or arm; muscular or joint pain in foot or leg; problems related to stomach or digestion; problems related to a skin condition; severe headaches; diabetes
	Health problems (on showcard) hampering daily activities in the last 12 months
	Currently have cancer; previously had cancer
Childhood conditions	Serious conflict between the people living in household when growing up
	Severe financial difficulties when growing up.
Quality of housing	Quality of housing any problems (as listed on showcard) with accommodation
Working conditions	Exposure in any job to: vibrations from hand tools or machinery; tiring or painful positions; manually lifting or moving people; manually carrying or moving heavy loads
	Exposure in any job to: very loud noise; very hot temperatures; very cold temperatures; radiation such as X-rays; handling, breathing in or being in contact with chemical products, vapours or substances; breathing in other types of smoke, fumes, powder or dust

**Table 5a:** Non-communicable diseases among European men (95% CIs)

Country		Heart	HBP	Breath	Allergy	Back pain	Arm pain	Leg pain	Stomach	Skin	Headache	Diabetes	1 of these	2 or more	Cancer pres.	Cancer prev.
ESS pooled	M	5.4	12.2	6.4	10.6	35.5	17.3	18.9	12.3	7.1	9.8	3.5	28.1	37.9	2.7	6.5
		(5.0–5.8)	(11.6–12.8)	(5.9–6.8)	(10.1–11.2)	(34.6–36.4)	(16.6–18.0)	(18.1–19.6)	(11.7–13.0)	(6.7–7.6)	(9.2–10.4)	(3.2–3.9)	(27.3–29.0)	(37.0–38.8)	(2.5–3.1)	(6.0–7.0)
Greece^a^	M	3.7	10.3	2.3	6.4	11.9	4.3	7.2	6.7	1.9	3.8	2.0	25.1	13.1	0.7	0.7
		(1.9–7.1)	(7.2–14.6)	(1.1–5.1)	(4.1–9.7)	(8.6–16.2)	(2.5–7.2)	(4.7–10.8)	(4.1–10.7)	(0.9–4.0)	(2.0–7.1)	(0.9–4.1)	(20.4–30.6)	(9.6–17.6)	(0.2–2.6)	(0.2–2.5)
Denmark	M	5.6	14.8	10.3	20.2	50.7	24.6	23.5	14.9	9.4	8.6	5.3	29.9	54.2	0.4	5.6
		(3.8–8.1)	(12.0–18.1)	(7.8–13.6)	(16.8–24.0)	(46.2–55.2)	(8.9–16.1)	(19.8–27.5)	(11.9–18.5)	(7.2–12.1)	(6.4–11.5)	(3.6–7.9)	(25.9–34.1)	(49.6–58.7)	(0.1–1.5)	(3.9–8.0)
Finland	M	6.1	16.1	9.5	18.4	50.4	25.7	30.0	16.7	15.8	14.5	4.7	29.5	57.5	0.7	3.2
		(4.6–7.9)	(13.8–18.8)	(7.4–12.1)	(15.7–21.5)	(46.5–54.2)	(22.5–22.9)	(26.6–33.7)	(14.0–19.8)	(13.2–18.8)	(12.0–17.5)	(3.5–6.3)	(26.2–33.1)	(53.7–61.2)	(0.3–1.6)	(2.1–4.8)
Norway	M	4.2	11.6	7.2	17.4	41.4	22.0	21.2	14.7	11.0	6.8	1.8	32.8	45.6	1.4	5.1
		(2.8–6.2)	(9.2–14.7)	(5.1–10.0)	(14.4–20.9)	(37.2–45.8)	(18.6–25.8)	(17.8–24.9)	(11.8–18.1)	(8.6–14.1)	(4.9–9.5)	(0.9–3.4)	(28.9–36.8)	(41.3–49.9)	(0.6–2.9)	(3.8–6.9)
Sweden	M	2.8	10.7	6.3	16.7	45.3	23.9	23.7	17.6	7.8	8.1	2.5	31.4	46.3	1.0	4.1
		(1.7–4.4)	(8.4–13.5)	(4.5–8.7)	(13.8–20.0)	(41.2–49.4)	(20.6–27.7)	(20.4–27.5)	(14.6–21.1)	(5.8–10.2)	(6.1–10.7)	(1.5–4.3)	(27.7–35.4)	(42.3–50.5)	(0.4–2.4)	(2.7–6.0)
Austria	M	5.1	11.3	4.5	7.9	28.4	10.9	12.5	8.0	5.8	9.4	2.2	22.0	28.5	3.2	9.4
		(3.7–7.1)	(8.9–14.3)	(3.0–6.6)	(5.8–10.7)	(24.8–32.2)	(8.7–13.7)	(10.1–15.5)	(5.9–10.8)	(4.1–8.1)	(7.3–12.1)	(1.3–3.9)	(18.7–25.7)	(24.9–32.4)	(2.0–5.0)	(7.2–12.1)
Belgium	M	5.8	10.6	7.6	11.0	48.1	22.9	24.0	15.1	7.0	10.1	3.6	31.5	45.9	1.8	4.3
		(4.2–7.8)	(8.5–13.1)	(5.8–9.9)	(8.8–13.7)	(44.2–52.1)	(19.8–26.4)	(20.8–27.5)	(12.5–18.1)	(5.2–9.2)	(8.0–12.7)	(2.4–5.3)	(27.2–36.2)	(42.0–49.8)	(1.0–3.1)	(3.0–6.2)
France	M	4.6	8.5	9.3	11.9	48.4	26.1	27.0	18.1	9.6	17.8	3.9	31.5	51.8	2.0	5.6
		(3.1–6.8)	(6.6–10.9)	(6.9–12.4)	(9.3–15.1)	(43.8–53.0)	(22.3–30.3)	(23.0–31.5)	(14.8–21.9)	(7.1–12.8)	(14.5–21.6)	(2.6–6.0)	(28.0–35.3)	(47.1–56.4)	(1.2–3.6)	(3.8–8.0)
Germany	M	8.4	17.5	9.0	15.7	51.2	19.5	22.4	19.7	12.8	18.2	3.7	26.8	55.7	1.2	3.7
		(6.7–10.4)	(15.3–20.0)	(7.2–11.2)	(13.5–18.3)	(47.8–54.6)	(17.0–22.3)	(19.8–25.4)	(17.1–22.6)	(10.7–15.4)	(15.7–21.1)	(2.8–4.9)	(23.8–29.9)	(52.4–59.1)	(0.7–2.1)	(2.7–5.1)
Ireland	M	2.3	7.0	5.0	4.1	20.4	8.5	12.2	7.1	4.6	3.6	1.6	22.3	20.7	4.1	6.8
		(1.5–3.6)	(5.4–9.1)	(3.6–7.0)	(2.9–5.9)	(17.3–23.8)	(6.5–11.0)	(9.8–15.0)	(5.4–9.4)	(3.2–6.6)	(2.4–5.4)	(0.9–2.7)	(19.2–25.8)	(17.7–24.0)	(2.8–5.9)	(5.1–8.9)
Netherlands	M	6.6	11.6	6.4	13.6	36.0	17.6	20.3	9.8	7.2	9.2	4.3	31.0	41.0	1.5	2.9
		(4.6–9.4)	(9.1–14.6)	(4.6–8.9)	(10.6–17.4)	(31.8–40.4)	(14.4–21.3)	(16.7–24.5)	(7.5–12.8)	(5.3–9.8)	(6.9–12.1)	(3.0–6.2)	(26.9–35.5)	(36.5–45.7)	(0.8–2.8)	(1.8–4.6)
Switzerland	M	4.2	8.9	5.2	10.6	37.8	16.6	23.4	14.0	8.1	13.6	2.2	33.0	40.7	4.8	9.5
		(2.8–6.3)	(6.9–11.5)	(3.6–7.5)	(8.3–13.5)	(33.8–42.0)	(13.7–20.0)	(19.9–27.2)	(11.3–17.3)	(6.0–10.7)	(11.0–16.7)	(1.2–3.8)	(29.1–37.1)	(36.6–44.9)	(3.4–6.9)	(7.3–12.2)
UK	M	3.9	12.8	10.3	9.6	36.5	20.9	24.7	15.7	9.3	9.1	4.5	31.2	40.8	1.2	4.7
		(2.7–5.6)	(10.4–15.5)	(7.9–13.3)	(7.3–12.6)	(32.6–40.7)	(17.7–24.6)	(21.2–28.7)	(12.8–19.0)	(7.0–12.4)	(6.7–12.2)	(3.1–6.4)	(27.3–35.3)	(36.7–45.1)	(0.6–2.2)	(3.2–6.8)
Czech Republic	M	3.3	11.1	2.8	5.9	18.5	7.3	9.0	5.8	4.9	5.0	3.4	27.9	17.8		
		(2.1–5.0)	(9.0–13.7)	(1.8–4.4)	(4.3–8.1)	(15.6–21.7)	(5.5–9.7)	(6.9–11.6)	(4.2–7.9)	(3.4–6.9)	(3.5–7.0)	(2.2–5.2)	(24.5–31.5)	(15.0–21.0)		
Estonia	M														4.1	9.0
															(2.7–6.0)	(6.9–11.6)
Hungary	M	4.6	14.3	3.2	4.2	12.8	6.7	8.6	4.2	0.8	6.6	3.7	18.1	19.1	9.9	15.1
		(3.2–6.7)	(11.7–17.5)	(2.0–5.1)	(2.8–6.3)	(10.1–15.9)	(4.9–9.1)	(6.5–11.4)	(2.7–6.3)	(0.3–2.2)	(4.7–9.1)	(2.4–5.6)	(15.9–21.7)	(16.1–22.6)	(7.7–12.8)	(12.3–18.4)
Lithuania	M	8.3	12.4	2.6	3.0	20.9	6.2	7.7	7.6	1.0	4.1	2.5	25.4	19.7	4.2	12.1
		(6.2–10.9)	(9.9–15.5)	(1.5–4.6)	(1.8–4.9)	(17.4–24.9)	(4.5–8.6)	(5.8–10.3)	(5.4–10.5)	(0.5–2.2)	(2.7–6.2)	(1.4–4.6)	(21.6–29.7)	(16.7–23.2)	(2.8–6.4)	(9.5–15.3)
Poland	M	9.4	13.7	2.4	8.0	30.7	20.1	17.5	10.9	4.0	7.8	2.0	28.0	33.9	2.4	4.6
		(7.3–12.0)	(11.1–16.8)	(1.4–4.1)	(6.1–10.5)	(27.0–34.7)	(17.0–23.7)	(14.6–20.9)	(8.6–13.7)	(2.7–5.9)	(5.8–10.4)	(1.1–3.7)	(24.3–32.0)	(30.1–37.9)	(1.4–4.1)	(3.2–6.8)
Slovenia	M	5.5	15.2	5.4	8.6	35.9	19.5	15.8	13.1	3.8	8.6	2.6	38.4	33.6	1.6	3.7
		(3.8–7.8)	(12.1–18.8)	(3.6–8.1)	(6.2–11.8)	(31.1–41.0)	(15.7–23.9)	(12.7–19.6)	(10.0–17.0)	(2.3–6.4)	(6.0–12.2)	(1.6–4.1)	(33.5–43.5)	(29.0–38.5)	(0.8–3.3)	(2.4–5.7)
Israel	M	5.5	10.2	6.5	5.9	20.7	12.8	10.8	6.4	5.1	8.9	7.6	18.7	24.9	7.4	12.6
		(3.9–7.6)	(7.9–13.0)	(4.5–9.1)	(4.3–8.1)	(17.5–24.2)	(10.2–15.9)	(8.5–13.7)	(4.6–8.9)	(3.6–7.3)	(6.6–11.8)	(5.5–10.3)	(15.7–22.1)	(21.5–28.6)	(5.4–10.1)	(10.0–15.8)
Portugal	M	5.4	16.1	8.1	18.2	61.4	27.5	32.4	21.5	7.2	20.6	5.4	39.2	60.8	1.2	3.7
		(3.2–8.9)	(12.2–20.8)	(5.1–12.5)	(13.6–23.9)	(54.6–67.7)	(22.1–33.6)	(26.3–39.3)	(16.7–27.3)	(4.5–11.3)	(15.5–26.9)	(3.1–9.3)	(32.8–46.1)	(53.9–67.2)	(0.4–3.3)	(2.0–6.8)
Spain	M	4.6	11.0	6.5	13.0	36.4	19.8	20.8	13.5	6.6	10.0	3.5	32.9	38.2	1.1	3.8
		(3.3–6.3)	(9.0–13.5)	(5.0–8.6)	(10.7–15.7)	(32.9–40.0)	(17.0–22.8)	(17.9–24.0)	(11.1–16.3)	(4.9–8.7)	(8.0–12.5)	(2.4–5.1)	(29.5–36.4)	(34.7–41.9)	(0.6–2.3)	(2.7–5.5)

**Table 5b:** 

Country		Heart	HBP	Breath	Allergy	Back pain	Arm pain	Leg pain	Stomach	Skin	Headache	Diabetes	1 of these	2 or more	Cancer pres.	Cancer prev.
ESS pooled	F	6.4	11.4	7.9	14.0	41.7	20.0	20.1	17.9	9.6	20.2	2.9	24.6	46.7	3.8	9.0
		(6.0–6.9)	(10.9–12.0)	(7.4–8.4)	(13.4–14.6)	(40.8–42.6)	(19.3–20.7)	(19.4–20.8)	(17.2–18.6)	(9.0–10.1)	(19.4–20.9)	(2.6–3.2)	(23.8–25.4)	(45.8–47.7)	(3.4–4.1)	(8.5–9.6)
Greece^a^	F	3.3	9.3	4.0	10.5	17.9	12.1	12.4	11.2	3.2	11.9	3.6	25.1	23.4	2.6	5.4
		(1.7–6.5)	(6.5–13.2)	(2.2–7.2)	(7.6–14.3)	(14.2–22.4)	(8.9–16.1)	(9.2–16.4)	(8.0–15.5)	(1.8–5.7)	(8.7–16.0)	(2.0–6.6)	(20.6–30.3)	(19.1–28.4)	(1.2–5.3)	(3.2–8.9)
Denmark	F	4.7	10.2	9.1	19.4	54.0	26.0	30.4	22.7	16.5	20.9	3.8	24.5	59.9	3.2	10.3
		(3.1–7.1)	(7.9–13.2)	(6.7–12.2)	(16.0–23.4)	(49.3–58.6)	(22.3–30.1)	(26.3–34.9)	(18.9–27.0)	(13.3–20.3)	(17.2–25.0)	(2.3–6.2)	(20.8–28.7)	(55.4–64.3)	(1.9–5.4)	(7.9–13.5)
Finland	F	4.6	13.7	12.4	22.3	60.7	25.3	29.0	29.4	22.6	27.7	3.6	21.6	69.1	1.7	5.1
		(3.3–6.6)	(11.4–16.3)	(10.0–15.2)	(19.3–25.7)	(56.9–64.4)	(22.2–28.8)	(25.7–32.6)	(26.0–33.0)	(19.5–26.1)	(24.4–31.4)	(2.4–5.2)	(18.6–25.0)	(65.4–72.6)	(1.0–2.9)	(3.8–6.9)
Norway	F	4.7	9.7	14.6	24.3	52.3	31.8	29.4	22.9	9.7	18.8	1.6	23.2	60.7	0.5	7.3
		(2.9–7.6)	(7.1–13.1)	(11.4–18.5)	(20.4–28.7)	(47.6–57.0)	(27.5–36.4)	(25.1–34.1)	(19.1–27.3)	(7.4–12.7)	(15.2–22.9)	(0.6–4.4)	(19.5–27.3)	(56.0–65.2)	(0.2–1.5)	(5.3–9.9)
Sweden	F	5.8	12.3	10.2	20.9	52.3	27.1	23.3	27.4	10.4	19.9	2.5	25.7	56.6	1.8	8.7
		(3.9–8.5)	(9.8–15.4)	(8.0–12.9)	(17.5–24.7)	(48.1–56.5)	(23.5–31.0)	(19.9–27.2)	(23.8–31.4)	(8.0–13.4)	(16.6–23.7)	(1.5–4.2)	(22.2–29.5)	(52.4–60.7)	(1.0–3.2)	(6.6–11.6)
Austria	F	4.8	9.7	5.3	10.5	33.5	13.9	13.0	13.4	8.5	15.6	1.5	19.0	35.6	1.8	9.5
		(3.3–7.0)	(7.7–12.1)	(3.7–7.4)	(8.2–13.3)	(29.8–37.3)	(11.3–16.9)	(10.5–16.0)	(10.7–16.5)	(6.6–11.1)	(12.9–18.8)	(0.8–3.0)	(16.0–22.4)	(31.9–39.5)	(1.1–3.1)	(7.4–12.1)
Belgium	F	6.4	14.9	9.2	16.7	54.3	26.9	25.7	26.4	8.9	25.8	1.6	24.5	60.6	2.4	7.7
		(4.8–8.6)	(12.4–17.7)	(7.2–11.8)	(14.0–19.8)	(50.4–58.1)	(23.6–30.4)	(22.4–29.2)	(23.1–29.9)	(6.9–11.4)	(22.6–29.4)	(0.9–2.8)	(21.3–28.1)	(56.7–64.4)	(1.4–3.9)	(5.9–10.0)
France	F	6.0	8.9	11.8	16.4	58.0	33.8	23.6	21.1	10.4	34.9	3.0	23.5	65.1	3.4	8.1
		(4.4–8.1)	(6.9–11.3)	(9.1–15.1)	(13.4–20.0)	(53.6–62.2)	(29.7–38.2)	(20.0–27.7)	(17.7–25.0)	(7.9–13.6)	(30.7–39.4)	(1.9–4.7)	(20.0–27.5)	(60.7–69.2)	(2.1–5.5)	(5.9–11.0)
Germany	F	10.9	15.0	9.9	19.3	61.3	22.2	22.3	25.5	15.3	31.9	3.0	21.8	66.4	2.3	7.7
		(9.0–13.2)	(12.9–17.4)	(8.1–12.2)	(16.8–22.1)	(58.0–64.5)	(19.6–25.1)	(19.8–25.0)	(22.7–28.6)	(13.3–17.8)	(28.9–35.1)	(2.1–4.3)	(19.2–24.7)	(63.1–69.4)	(1.5–3.5)	(6.2–9.6)
Ireland	F	2.4	6.7	7.3	6.8	22.7	10.5	11.6	10.4	6.9	7.4	1.5	24.5	23.7	4.1	7.7
		(1.5–3.8)	(5.1–8.7)	(5.6–9.4)	(5.1–9.0)	(1.99–25.8)	(8.6–12.9)	(9.6–14.0)	(8.4–12.7)	(5.2–9.1)	(5.8–9.5)	(0.9–2.6)	(21.6–27.7)	(20.8–26.9)	(2.8–5.9)	(6.1–9.6)
Netherlands	F	4.8	11.6	9.7	16.3	45.9	18.4	20.4	16.6	10.0	19.7	2.2	33.7	46.4	2.4	7.3
		(3.4–6.7)	(9.1–14.6)	(7.6–12.3)	(13.5–19.4)	(41.9–49.9)	(15.6–21.5)	(17.4–23.7)	(13.9–19.6)	(8.0–12.5)	(16.7–23.0)	(1.3–3.8)	(30.0–37.6)	(42.5–50.3)	(1.4–4.1)	(5.7–9.5)
Switzerland	F	5.5	7.4	7.2	14.8	45.4	18.8	21.4	18.9	11.0	23.2	1.7	31.2	48.0	7.8	15.5
		(3.8–7.8)	(5.5–9.9)	(5.3–9.8)	(12.0–18.1)	(41.2–49.6)	(15.7–22.2)	(18.2–25.0)	(15.8–22.4)	(8.6–13.9)	(19.9–27.0)	(0.9–3.2)	(27.3–35.3)	(43.8–52.2)	(5.9–10.4)	(12.7–18.8)
UK	F	5.0	12.3	11.2	15.1	39.0	19.8	25.5	17.0	14.1	19.9	3.2	28.1	48.7	3.0	8.1
		(3.7–6.8)	(10.1–14.9)	(9.1–13.7)	(12.6–18.0)	(35.4–42.6)	(17.3–22.7)	(22.4–28.8)	(14.5–19.8)	(11.7–17.0)	(17.1–23.0)	(2.2–4.6)	(24.7–31.8)	(45.0–52.5)	(1.9–4.8)	(6.3–10.4)
Czech Republic	F	2.8	8.5	4.2	11.1	25.2	8.9	10.1	7.2	4.5	12.9	4.0	28.8	24.8		
		(1.8–4.3)	(6.8–10.6)	(3.0–6.1)	(9.0–13.8)	(22.2–28.4)	(7.0–11.2)	(8.2–12.5)	(5.5–9.4)	(3.2–6.2)	(10.6–15.5)	(2.8–5.7)	(25.6–32.3)	(21.9–28.0)		
Estonia	F														4.9	9.6
															(3.5–6.7)	(7.7–12.0)
Hungary	F	7.7	14.9	3.6	8.0	14.5	10.5	11.2	6.6	3.9	14.5	3.8	16.5	24.4	9.8	15.6
		(5.9–10.0)	(12.5–17.6)	(2.3–5.5)	(6.1–10.5)	(12.0–17.4)	(8.4–13.1)	(9.0–13.9)	(4.9–8.9)	(2.6–5.7)	(11.9–17.5)	(2.6–5.6)	(13.9–19.6)	(21.3–27.7)	(7.8–12.4)	(13.0–18.6)
Lithuania	F	13.0	14.1	2.9	5.2	25.3	8.1	9.6	15.3	4.8	12.3	1.9	23.1	32.3	6.6	14.6
		(10.7–15.8)	(11.8–16.7)	(1.9–4.5)	(3.7–7.4)	(22.2–28.8)	(6.2–10.4)	(7.6–12.0)	(12.6–18.4)	(3.4–6.8)	(9.9–15.2)	(1.0–3.5)	(20.0–26.4)	(28.9–35.8)	(4.9–8.8)	(12.2–17.5)
Poland	F	12.1	12.8	4.4	13.0	37.1	22.0	18.5	16.1	5.1	20.7	3.1	24.1	45.5	3.9	8.7
		(9.8–14.9)	(10.5–15.5)	(3.0–6.3)	(10.6–15.9)	(33.3–41.0)	(18.9–25.4)	(15.6–21.8)	(13.3–19.4)	(3.5–7.3)	(17.6–24.2)	(2.0–4.7)	(20.7–27.7)	(41.5–49.5)	(2.6–5.8)	(6.6–11.2)
Slovenia	F	8.1	15.8	6.4	12.0	46.7	18.1	19.2	21.4	4.3	16.9	3.4	30.8	48.7	2.0	6.1
		(6.0–10.9)	(13.0–19.1)	(4.6–9.0)	(9.3–15.3)	(42.2–51.3)	(14.9–21.7)	(16.0–23.0)	(17.9–25.4)	(2.8–6.5)	(13.7–20.8)	(2.2–5.2)	(26.6–35.2)	(44.2–53.3)	(1.1–3.8)	(4.4–8.5)
Israel	F	4.0	8.3	5.4	5.6	22.2	9.9	14.3	9.8	4.6	11.0	6.2	20.2	25.7	7.5	10.4
		(2.8–5.7)	(6.5–10.5)	(3.9–7.5)	(4.2–7.4)	(19.3–25.4)	(7.9–12.2)	(11.9–17.2)	(7.9–12.2)	(3.3–6.5)	(9.0–13.4)	(4.6–8.2)	(17.4–23.3)	(22.7–29.0)	(5.7–9.9)	(8.3–13.0)
Portugal	F	10.6	16.4	11.3	25.1	60.7	45.9	36.9	22.5	6.6	39.7	5.3	22.2	77.8	3.3	9.7
		(7.6–14.7)	(12.6–21.2)	(8.0–15.8)	(19.8–31.1)	(54.3–66.7)	(39.5–52.3)	(31.5–42.6)	(17.8–28.0)	(4.4–9.7)	(33.5–46.1)	(3.4–8.0)	(17.6–27.6)	(72.4–82.4)	(1.8–6.0)	(7.0–13.4)
Spain	F	5.5	8.7	6.5	12.1	48.0	26.9	25.5	17.5	11.3	23.4	2.0	27.1	49.2	1.2	4.3
		(4.0–7.5)	(6.9–10.9)	(4.8–8.7)	(9.8–14.8)	(44.2–51.8)	(23.8–30.3)	(22.4–29.0)	(14.7–20.6)	(9.1–14.0)	(20.3–26.8)	(1.2–3.4)	(23.8–30.7)	(45.4–53.0)	(0.6–2.4)	(3.0–6.1)

Note: ^a^Sample consists of individuals living in Greece aged between 20 and 64. CIs, confidence intervals.

**Table 6:** Self-reported health (95% CIs)

Country		Poor/very poor health	Hampered by illness	Depressive symptoms	Overweight/obese
ESS pooled	M	4.3 (4.0–4.7)	19.3 (18.6–20.1)	9.5 (9.0–10.1)	58.5 (57.6–59.4)
	F	5.8 (5.4–6.2)	22.3 (21.6–23.0)	14.5 (13.9–15.1)	41.3 (40.5–42.2)
Greece	M	2.8 (1.4–5.6)	8.6 (5.8–12.7)	26.2 (21.3–31.8)	58.5 (52.6–64.2)
	F	6.0 (3.7–9.7)	13.7 (10.1–18.4)	37.1 (31.8–42.7)	41.8 (36.7–47.0)
Denmark	M	6.3 (4.4–8.9)	24.0 (20.3–28.1)	9.0 (6.7–11.9)	53.3 (48.9–57.7)
	F	5.7 (3.9–8.4)	31.9 (27.7–36.4)	13.1 (10.3–16.6)	38.1 (33.6–42.8)
Finland	M	2.6 (1.7–3.9)	25.0 (21.9–28.4)	6.3 (4.7–8.4)	61.0 (57.2–64.6)
	F	3.0 (1.8–5.0)	29.4 (26.0–33.0)	7.0 (5.2–9.5)	44.5 (40.7–48.3)
Norway	M	3.9 (2.4–6.2)	20.4 (17.1–24.2)	5.7 (3.8–8.4)	61.4 (57.2–65.5)
	F	11.1 (8.0–15.1)	30.2 (25.9–35.0)	9.2 (6.5–13.0)	39.8 (35.1–44.7)
Sweden	M	2.1 (1.2–3.7)	22.2 (19.0–25.8)	5.9 (4.3–8.1)	57.8 (53.8–61.6)
	F	4.7 (3.2–6.8)	31.7 (27.8–35.8)	14.3 (11.4–17.7)	43.4 (39.3–47.6)
Austria	M	3.5 (2.1–5.5)	17.1 (14.2–20.6)	8.8 (6.6–11.7)	56.9 (52.7–60.9)
	F	3.4 (2.2–5.2)	15.6 (13.0–18.7)	10.7 (8.4–13.5)	33.9 (30.3–37.7)
Belgium	M	3.8 (2.5–5.7)	19.6 (16.7–22.8)	6.3 (4.7–8.5)	53.2 (49.4–57.0)
	F	4.3 (3.0–6.2)	23.8 (20.6–27.3)	14.6 (12.0–17.6)	37.5 (33.8–41.3)
France	M	4.7 (3.2–6.8)	18.0 (14.7–21.8)	7.6 (5.4–10.7)	50.6 (46.2–55.1)
	F	6.5 (4.6–9.2)	21.8 (18.4–25.5)	15.3 (12.4–18.8)	37.2 (32.9–41.8)
Germany	M	6.9 (5.4–8.7)	25.3 (22.5–28.3)	9.6 (7.8–11.9)	59.7 (56.4–63.0)
	F	8.8 (7.0–11.0)	27.0 (24.2–30.0)	16.3 (14.0–19.0)	41.5 (38.2–44.8)
Ireland	M	1.6 (0.9–3.0)	14.2 (11.7–17.2)	5.6 (4.2–7.6)	56.7 (52.8–60.5)
	F	2.1 (1.4–3.2)	13.0 (10.9–15.5)	8.0 (6.3–10.0)	40.3 (36.9–43.8)
Netherlands	M	3.8 (2.5–5.8)	23.2 (19.7–27.0)	8.4 (6.2–11.1)	53.6 (49.1–58.1)
	F	4.5 (3.3–6.1)	28.6 (25.2–32.3)	7.9 (6.1–10.2)	40.3 (36.6–44.1)
Switzerland	M	2.2 (1.2–3.9)	15.6 (12.8–18.8)	6.0 (4.3–8.4)	51.4 (47.3–55.5)
	F	4.0 (2.7–6.1)	19.7 (16.6–23.3)	9.0 (6.9–11.8)	28.5 (24.9–32.4)
UK	M	5.1 (3.7–7.1)	19.7 (16.5–23.3)	11.5 (9.1–14.5)	62.0 (57.8–65.9)
	F	7.4 (5.7–9.5)	22.7 (19.8–25.8)	16.1 (13.6–18.8)	49.5 (45.6–53.5)
Czech Republic	M	2.5 (1.6–4.0)	17.8 (15.1–20.9)	17.5 (14.6–20.8)	67.3 (63.4–70.9)
	F	3.3 (2.2–4.8)	20.9 (18.2–23.9)	22.1 (19.2–25.3)	44.5 (41.1–48.0)
Estonia	M	6.9 (5.2–9.3)	20.8 (17.7–24.2)	12.6 (10.1–15.6)	56.4 (52.6–60.2)
	F	7.1 (5.5–9.2)	17.4 (14.9–20.2)	15.8 (13.3–18.6)	41.2 (37.9–44.5)
Hungary	M	7.2 (5.2–9.8)	17.6 (14.6–21.1)	15.0 (12.0–18.5)	68.3 (64.0–72.3)
	F	7.2 (5.4–9.6)	20.5 (17.7–23.7)	19.6 (16.6–23.0)	48.2 (44.4–52.0)
Lithuania	M	4.4 (3.0–6.6)	20.1 (17.0–23.6)	11.1 (8.5–14.4)	60.9 (56.4–65.1)
	F	5.3 (3.9–7.2)	23.5 (20.6–26.7)	15.1 (12.5–18.0)	45.9 (42.3–49.6)
Poland	M	4.3 (2.9–6.2)	19.9 (16.9–23.3)	10.8 (8.5–13.7)	62.6 (58.7–66.3)
	F	6.1 (4.5–8.2)	24.0 (20.9–27.5)	19.2 (16.3–22.6)	40.9 (37.2–44.7)
Slovenia	M	5.5 (3.7–7.9)	22.3 (18.7–26.3)	7.1 (5.0–10.0)	62.7 (57.7–67.4)
	F	6.2 (4.5–8.6)	29.8 (25.9–34.0)	10.5 (8.0–13.6)	44.1 (39.7–48.5)
Israel	M	5.3 (3.6–7.8)	17.3 (14.3–20.8)	10.3 (8.1–13.2)	56.8 (53.1–60.4)
	F	7.1 (5.5–9.3)	19.0 (16.4–21.9)	15.7 (13.2–18.6)	46.2 (42.8–49.7)
Portugal	M	4.3 (2.3–7.7)	15.5 (11.6–20.4)	13.2 (9.6–17.9)	55.9 (49.8–61.9)
	F	7.7 (5.4–10.9)	16.3 (12.6–20.9)	24.7 (20.3–29.8)	44.4 (38.9–50.1)
Spain	M	4.5 (3.3–6.2)	10.1 (8.2–12.5)	10.2 (8.2–12.6)	59.4 (55.9–62.7)
	F	8.6 (6.7–10.9)	11.2 (9.1–13.8)	19.5 (16.6–22.6)	34.8 (31.4–38.4)

Note: Sample consists of individuals living in Greece aged between 20 and 64. CIs, confidence intervals.

**Table 7:** Healthcare access and healthcare utilization (95% CIs)

Country		Unmet need: overall	Unmet need: waiting list	Unmet need: no appointment avail	Visited GP	Visited specialist	Used alternative treatment
ESS pooled	M	11.3 (10.7–11.9)	4.1 (3.7–4.5)	3.6 (3.3–4.0)	65.3 (64.4–66.2)	32.2 (31.4–33.1)	30.7 (29.8–31.5)
	F	15.1 (14.5–15.8)	5.2 (4.9–5.6)	5.8 (5.4–6.3)	75.6 (74.9–76.4)	44.3 (43.5–45.2)	41.2 (40.3–42.0)
Greece	M	15.4 (11.6–20.2)	6.2 (4.0–9.6)	5.0 (2.9–8.3)	11.8 (8.5–16.1)	37.7 (32.2–43.6)	8.1 (5.4–11.9)
	F	27.3 (22.5–32.7)	9.6 (6.5–14.1)	8.0 (5.6–11.4)	14.7 (11.2–19.0)	56.8 (51.2–62.2)	11.9 (8.8–15.8)
Denmark	M	7.0 (5.0–9.7)	2.0 (1.0–3.8)	2.6 (1.4–4.6)	73.8 (69.5–77.6)	34.1 (29.9–38.5)	39.6 (35.3–44.1)
	F	10.0 (7.4–13.4)	3.4 (2.0–5.8)	2.5 (1.3–4.5)	83.4 (79.8–86.5)	42.8 (38.2–47.5)	49.9 (45.3–54.6)
Finland	M	16.8 (14.1–20.0)	6.1 (4.5–8.3)	5.2 (3.7–7.2)	66.7 (63.0–70.2)	33.0 (29.6–36.7)	43.1 (39.4–46.9)
	F	22.3 (19.1–25.7)	6.8 (5.0–9.1)	9.8 (7.6–12.4)	70.6 (66.9–74.0)	48.4 (44.6–52.3)	59.1 (55.2–62.8)
Norway	M	12.3 (9.7–15.4)	4.7 (3.1–7.1)	5.4 (3.7–7.8)	72.2 (68.2–75.8)	22.9 (19.5–26.7)	36.9 (32.8–41.2)
	F	17.5 (14.1–21.6)	6.6 (4.6–9.4)	5.4 (3.5–8.2)	82.1 (78.5–85.3)	32.5 (28.3–37.1)	46.1 (41.3–50.9)
Sweden	M	9.8 (7.6–12.7)	2.0 (1.1–3.6)	2.5 (1.5–4.1)	47.7 (43.6–51.8)	24.8 (21.4–28.5)	40.9 (36.9–45.1)
	F	13.2 (10.5–16.5)	2.1 (1.2–3.8)	3.2 (2.0–5.0)	59.4 (55.3–63.5)	38.4 (34.5–42.5)	50.5 (46.4–54.7)
Austria	M	5.3 (3.7–7.6)	1.9 (1.1–3.3)	3.1 (1.9–5.1)	67.9 (63.7–71.8)	34.0 (30.2–38.1)	34.2 (30.4–38.2)
	F	6.3 (4.6–8.5)	1.7 (0.9–3.2)	3.2 (2.0–5.0)	79.5 (76.1–82.6)	55.7 (51.7–59.6)	48.4 (44.4–52.4)
Belgium	M	10.4 (8.2–13.0)	2.8 (1.8–4.5)	2.2 (1.3–3.7)	73.9 (70.3–77.2)	32.4 (28.9–36.1)	33.0 (29.4–36.8)
	F	13.4 (11.0–16.4)	4.0 (2.7–5.8)	1.7 (0.9–3.0)	84.9 (81.9–87.4)	50.8 (46.9–54.7)	40.8 (37.0–44.7)
France	M	17.0 (13.8–20.8)	4.0 (2.6–6.2)	3.3 (2.1–5.3)	76.6 (72.3–80.5)	37.4 (33.0–42.0)	38.3 (33.9–43.0)
	F	26.7 (23.0–30.7)	6.3 (4.5–8.8)	8.2 (6.2–10.9)	83.4 (79.8–86.5)	53.6 (49.1–58.1)	52.2 (47.7–56.7)
Germany	M	15.4 (13.2–18.0)	5.0 (3.7–6.7)	5.5 (4.2–7.2)	77.0 (74.0–79.7)	51.0 (47.6–54.4)	40.8 (37.5–44.1)
	F	22.5 (19.9–25.4)	7.3 (5.8–9.2)	9.0 (7.3–11.0)	81.7 (79.0–84.2)	69.4 (66.2–72.5)	56.9 (53.6–60.2)
Ireland	M	5.7 (4.2–7.9)	1.8 (1.0–3.2)	1.5 (0.8–2.8)	56.8 (52.9–60.7)	13.2 (10.8–16.1)	23.0 (19.9–26.5)
	F	8.7 (6.8–10.9)	3.2 (2.2–4.7)	3.0 (2.0–4.3)	70.4 (67.1–73.6)	17.3 (14.8–20.0)	32.0 (28.8–35.5)
Netherlands	M	4.6 (2.9–7.1)	1.1 (0.4–2.9)	0.7 (0.2–3.2)	59.7 (55.1–64.2)	34.4 (30.2–38.8)	34.9 (30.5–39.5)
	F	4.6 (3.2–6.4	0.7 (0.3–1.6)	0.7 (0.3–1.8)	73.6 (69.9–77.0)	41.9 (38.1–45.9)	40.2 (36.3–44.1)
Switzerland	M	5.7 (4.0–8.1)	0.4 (0.1–1.6)	1.0 (0.4–2.4)	60.6 (56.4–64.6)	35.3 (31.4–39.4)	42.4 (38.2–46.6)
	F	9.0 (6.9–11.8)	1.2 (0.5–2.6)	1.9 (1.1–3.6)	70.2 (66.3–73.9)	44.8 (40.6–49.0)	59.2 (54.9–63.2)
UK	M	12.8 (10.3–15.9)	2.4 (1.4–4.1)	5.8 (4.1–8.0)	66.5 (62.3–70.4)	25.7 (22.3–29.5)	26.6 (22.9–30.6)
	F	17.4 (14.8–20.2)	3.4 (2.3–5.1)	12.7 (10.5–15.2)	75.5 (72.0–78.7)	34.1 (30.6–37.6)	34.3 (30.8–37.9)
Czech Republic	M	6.0 (4.4–8.1)	0.7 (0.3–1.5	1.2 (0.6–2.2)	66.3 (62.4–70.1)	24.5 (21.3–28.1)	24.9 (21.5–28.6)
	F	5.4 (4.0–7.2)	1.6 (0.9–2.8)	0.9 (0.4–2.0)	71.7 (68.3–74.9)	34.4 (31.0–37.9)	35.1 (31.7–38.7)
Estonia	M	14.1 (11.5–17.1)	8.1 (6.1–10.6)	4.0 (2.6–6.0)	60.4 (56.4–64.2)	36.8 (33.0–40.7)	31.2 (27.6–35.0)
	F	21.6 (18.7–24.7)	11.4 (9.3–13.9)	8.8 (7.0–11.0)	75.5 (72.2–78.5)	57.0 (53.4–60.5)	51.5 (47.9–55.0)
Hungary	M	4.3 (2.9–6.4)	2.0 (1.1–3.7)	1.2 (0.5–2.8)	49.4 (45.1–53.7)	20.4 (17.3–23.9)	11.0 (8.7–14.0)
	F	5.9 (4.3–8.0)	3.0 (1.9–4.6)	2.2 (1.3–3.7)	62.9 (59.2–66.5)	28.9 (25.5–32.6)	15.3 (12.8–18.3)
Lithuania	M	8.4 (6.0–11.5)	2.9 (1.7–5.0)	3.8 (2.3–6.2)	45.6 (41.2–50.1)	15.1 (12.0–18.9)	25.5 (21.6–29.9)
	F	14.8 (12.2–17.9)	5.3 (3.7–7.6)	6.6 (4.8–9.1)	66.5 (62.7–70.0)	27.1 (23.7–30.8)	44.9 (41.1–48.7)
Poland	M	19.6 (16.5–23.2)	10.3 (8.1–13.1)	7.6 (5.6–10.1)	59.9 (55.7–63.9)	36.0 (32.1–40.1)	15.6 (12.9–18.9)
	F	26.4 (22.9–30.1)	13.1 (10.5–16.1)	11.8 (9.5–14.7)	71.3 (67.4–74.8)	46.7 (42.7–50.7)	19.9 (17.0–23.3)
Slovenia	M	8.1 (5.7–11.3)	3.9 (2.4–6.5)	0.2 (0.0–1.5)	73.9 (69.2–78.2)	31.8 (27.3–36.7)	31.9 (27.3–36.9)
	F	7.7 (5.6–10.4)	3.9 (2.5–5.9)	0.7 (0.2–2.0)	77.7 (73.6–81.3)	40.6 (36.2–45.2)	38.5 (34.1–43.1)
Israel	M	15.8 (13.0–19.0)	10.6 (8.2–13.5)	7.5 (5.6–10.1)	72.1 (68.5–75.4)	49.3 (45.5–53.3)	20.7 (17.6–24.2)
	F	21.9 (19.1–25.1)	11.7 (9.6–14.2)	11.6 (9.5–14.2)	84.2 (81.4–86.6)	61.6 (58.1–65.0)	26.7 (23.7–30.0)
Portugal	M	20.7 (15.9–26.6)	6.5 (3.7–11.1)	5.1 (2.9–8.7)	74.9 (69.4–79.6)	34.5 (29.1–40.5)	22.1 (17.4–27.6)
	F	18.5 (14.6–23.2)	3.5 (2.0–5.9)	5.0 (3.2–7.7)	81.9 (77.1–86.0)	44.0 (38.5–49.6)	25.3 (20.7–30.5)
Spain	M	14.8 (12.3–17.7)	4.9 (3.5–6.8)	4.1 (2.8–5.9)	71.3 (67.8–74.5)	39.5 (36.0–43.2)	29.0 (25.7–32.4)
	F	16.5 (13.8–19.6)	6.2 (4.6–8.3)	4.5 (3.1–6.4)	80.6 (77.3–83.4)	51.4 (47.6–55.2)	36.8 (33.2–40.6)

Note: Sample consists of individuals living in Greece aged between 20 and 64. CIs, confidence intervals.

**Table 8a:** Risk behaviour among European men (95% CIs)

Country		Smoking current	Smoke previous	Cigs ≥ 20 per day	Alcohol > once per week	Units on weekday (mean)	Units on weekend (mean)	Binge at least weekly	Physical activity on 3–4 days
ESS pooled	M	34.3	48.1	38.8	29.8	4.5	8.6	24.0	22.0
		(33.4–35.2)	(47.0–49.3)	(37.2–40.4)	(29.0–30.6)	(4.4–4.6)	(8.4–8.7)	(23.2–24.9)	(21.3–22.8)
Greece	M	42.7	34.9	50.5	26.1	6.9	7.8	42.4	20.1
		(37.0–48.5)	(28.5–42.0)	(41.6–59.3)	(21.2–31.6)	(5.5–8.2)	(6.6–9.0)	(36.0–49.1)	(15.6–25.5)
Denmark	M	31.1	55.6	42.4	35.7	4.3	10.0	18.3	21.0
		(27.0–35.5)	(50.1–60.9)	(34.2–51.0)	(31.7–40.0)	(3.8–4.9)	(9.2–10.7)	(15.1–22.1)	(17.6–24.8)
Finland	M	31.1	60.2	32.9	18.9	4.1	9.9	23.7	31.6
		(27.6–34.9)	(55.8–64.4)	(26.7–39.8)	(16.2–22.0)	(3.7–4.6)	(9.2–10.6)	(20.6–27.1)	(28.2–35.3)
Norway	M	23.5	63.8	23.6	20.1	5.0	10.7	22.0	27.8
		(19.9–27.5)	(58.3–69.0)	(16.1–33.3)	(16.9–23.7)	(4.4–5.5)	(9.9–11.4)	(18.5–26.0)	(24.1–31.9)
Sweden	M	15.4	77.2	17.2	21.8	4.4	8.8	27.9	24.8
		(12.5–18.9)	(72.4–81.3)	(12.5–23.0)	(18.6–25.4)	(3.9–4.9)	(8.2–9.4)	(24.1–32.0)	(21.4–28.6)
Austria	M	34.9	42.2	56.9	37.4	4.4	7.0	26.4	24.8
		(31.0–38.9)	(37.1–47.5)	(49.8–63.7)	(33.5–41.4)	(3.9–4.8)	(6.4–7.5)	(22.7–30.6)	(21.2–28.7)
Belgium	M	30.8	50.0	39.8	42.4	3.9	6.8	25.8	18.8
		(27.3–34.6)	(45.0–55.0)	(33.1–46.8)	(38.6–46.3)	(3.5–4.4)	(6.3–7.3)	(22.3–29.8)	(16.0–22.0)
France	M	36.5	46.9	36.6	40.3	2.6	5.3	12.6	18.8
		(32.1–41.3)	(41.3–52.7)	(29.9–43.7)	(36.0–44.9)	(2.3–2.8)	(4.8–5.9)	(9.7–16.3)	(15.5–22.7)
Germany	M	39.5	47.1	39.5	35.4	3.3	6.3	17.6	23.0
		(36.2–42.9)	(43.1–51.1)	(34.0–45.2)	(32.5–38.5)	(3.1–3.6)	(5.9–6.7)	(15.1–20.3)	(20.3–26.0)
Ireland	M	27.5	46.1	41.6	24.2	6.7	13.5	48.3	26.8
		(24.1–31.1)	(40.8–51.5)	(34.4–49.0)	(20.9–27.8)	(6.1–7.3)	(12.8–14.3)	(43.8–52.7)	(23.5–30.5)
Netherlands	M	35.0	49.9	24.9	44.3	3.0	6.4	25.0	23.2
		(30.7–39.7)	(44.5–55.4)	(18.7–32.2)	(39.8–48.9)	(2.7–3.4)	(5.8–7.0)	(20.8–29.7)	(19.6–27.3)
Switzerland	M	32.4	47.0	40.7	37.5	3.2	5.4	15.8	22.2
		(28.5–36.4)	(41.6–52.4)	(33.6–48.1)	(33.5–41.6)	(2.8–3.5)	(4.9–5.9)	(12.9–19.4)	(18.9–25.9)
UK	M	26.7	55.0	25.2	38.2	6.1	10.3	39.8	19.7
		(23.0–30.8)	(49.4–60.5)	(19.1–32.6)	(34.2–42.3)	(5.4–6.7)	(9.5–11.1)	(35.3–44.6)	(16.5–23.4)
Czech Republic	M	37.9	38.9	29.0	27.1	6.9	11.0	25.2	23.6
		(34.2–41.8)	(34.0–44.1)	(23.4–35.2)	(23.7–30.7)	(6.3–7.6)	(10.2–11.8)	(21.5–29.4)	(20.3–27.2)
Estonia	M	40.8	46.6	41.2	21.5	4.3	9.8	21.3	21.5
		(37.0–44.8)	(42.1–51.2)	(35.2–47.5)	(18.4–25.0)	(3.7–4.8)	(9.0–10.6)	(18.0–25.1)	(18.4–25.0)
Hungary	M	41.6	32.9	44.1	22.0	6.0	11.4	15.6	16.7
		(37.3–46.1)	(27.9–38.3)	(37.5–50.9)	(18.6–25.8)	(5.3–6.7)	(10.3–12.4)	(12.4–19.6)	(13.7–20.3)
Lithuania	M	48.7	40.1	36.1	23.0	6.9	14.2	32.1	19.3
		(44.0–53.4)	(35.1–45.4)	(30.1–42.6)	(19.3–27.1)	(6.1–7.7)	(13.2–15.2)	(27.6–36.9)	(15.8–23.3)
Poland	M	39.9	45.8	54.5	19.7	5.4	9.2	17.8	15.9
		(35.8–44.1)	(41.0–50.7)	(48.1–60.8)	(16.6–23.3)	(4.7–6.0)	(8.6–9.9)	(14.6–21.4)	(13.1–19.3)
Slovenia	M	33.0	48.6	50.7	25.7	3.4	4.8	11.7	16.3
		(28.4–38.0)	(42.7–54.7)	(41.8–59.6)	(21.6–30.3)	(3.0–3.8)	(4.3–5.3)	(8.6–15.6)	(12.8–20.5)
Israel	M	34.7	33.7	53.5	11.7	2.9	5.0	26.5	20.6
		(31.0–38.5)	(28.7–39.0)	(47.0–59.9)	(9.4–14.5)	(2.5–3.4)	(4.5–5.6)	(21.8–31.8)	(17.5–24.1)
Portugal	M	37.6	47.0	39.6	46.6	4.0	5.1	24.0	16.9
		(32.0–43.5)	(40.1–54.1)	(30.5–49.5)	(40.8–52.6)	(3.3–4.7)	(4.5–5.6)	(18.6–30.4)	(12.7–22.1)
Spain	M	36.5	44.7	30.9	37.7	2.1	5.1	13.7	20.1
		(33.1–40.1)	(40.5–48.9)	(35.6–36.6)	(34.3–41.2)	(1.9–2.3)	(4.8–5.5)	(11.2–16.5)	(17.3–23.2)

**Table 8b:** Risk behaviour among European women (95% CIs)

Country		Smoking current	Smoke previous	Cigs ≥ 20 per day	Alcohol > once per week	Units on weekday (mean)	Units on weekend (mean)	Binge at least weekly	Physical activity on 3–4 days
ESS pooled	F	24.5	53.7	22.1	13.9	2.6	5.1	12.4	22.0
		(23.7–25.2)	(52.5–54.9)	(20.7–23.7)	(13.3–14.5)	(2.6–2.7)	(5.0–5.2)	(11.7–13.0)	(21.3–22.7)
Greece	F	42.6	22.3	46.4	11.5	4.7	5.1	24.3	19.1
Denmark		(37.2–48.2)	(16.6–29.2)	(38.0–55.0)	(8.4–15.5)	(3.8–5.7)	(4.2–6.0)	(19.0–30.6)	(15.3–23.7)
	F	23.0	64.6	22.4	20.9	3.1	6.5	8.7	25.6
Finland		(19.3–27.2)	(58.8–69.9)	(15.9–30.5)	(17.6–24.7)	(2.6–3.6)	(5.8–7.1)	(6.3–11.9)	(21.7–29.9)
	F	23.3	64.3	17.2	7.2	2.4	6.0	8.1	28.7
Norway		(20.1–26.9)	(59.6–68.8)	(11.6–24.6)	(5.5–9.3)	(2.2–2.7)	(5.6–6.5)	(6.1–10.5)	(25.3–32.3)
	F	18.0	73.6	18.4	10.7	2.9	6.2	10.0	27.3
Sweden		(14.5–22.1)	(68.1–78.5)	(10.7–29.8)	(8.2–13.9)	(2.5–3.3)	(5.7–6.8)	(7.4–13.3)	(23.4–31.5)
	F	15.0	76.3	9.5	11.7	2.8	5.7	19.2	27.1
		(12.3–18.3)	(71.6–80.5)	(5.0–17.3)	(9.4–14.4)	(2.5–3.1)	(5.3–6.0)	(16.1–22.7)	(23.6–30.9)
Austria	F	29.9	43.5	39.1	14.5	2.7	4.5	11.4	27.1
		(26.4–33.7)	(38.2–49.1)	(32.6–46.0)	(11.9–17.6)	(2.3–3.1)	(4.0–4.9)	(8.8–14.6)	(23.7–30.9)
Belgium	F	25.9	51.1	31.7	25.2	2.1	4.1	14.5	19.8
		(22.6–29.6)	(45.6–56.5)	(25.0–39.4)	(22.1–28.7)	(1.9–2.3)	(3.7–4.5)	(11.7–17.8)	(16.9–23.1)
France	F	30.4	48.5	18.9	17.6	1.7	3.0	4.8	14.7
		(26.5–34.6)	(42.8–54.2)	(13.3–26.0)	(14.6–21.0)	(1.4–1.9)	(2.7–3.3)	(3.3–7.1)	(11.9–18.1)
Germany	F	31.9	49.5	19.5	16.3	2.0	3.7	11.9	23.2
		(28.8–35.1)	(45.3–53.8)	(15.3–24.4)	(14.1–18.9)	(1.8–2.1)	(3.5–4.0)	(9.8–14.4)	(20.5–26.2)
Ireland	F	24.5	49.6	30.0	11.9	4.1	8.5	33.3	29.4
		(21.6–27.7)	(44.5–54.8)	(24.0–36.8)	(9.8–14.4)	(3.6–4.5)	(8.0–9.0)	(29.6–37.1)	(26.2–32.8)
Netherlands	F	21.4	61.6	22.6	28.9	1.7	3.3	10.3	26.8
		(18.3–24.9)	(56.4–66.6)	(16.4–30.3)	(25.6–32.5)	(1.5–1.8)	(3.0–3.6)	(7.9–13.3)	(23.4–30.5)
Switzerland	F	27.3	51.1	19.4	19.1	1.8	3.4	6.7	21.5
		(23.7–31.2)	(45.5–56.6)	(13.8–26.5)	(16.1–22.6)	(1.6–2.0)	(3.0–3.7)	(4.7–9.4)	(18.3–25.2)
UK	F	23.4	55.2	18.3	26.7	3.7	6.9	31.1	24.3
		(20.5–26.7)	(50.3–60.0)	(13.8–23.9)	(23.6–30.0)	(3.2–4.1)	(6.2–7.5)	(27.4–35.1)	(21.2–27.7)
Czech Republic	F	22.2	46.8	12.5	9.3	4.8	7.3	12.7	23.4
		(19.3–25.4)	(41.1–52.6)	(8.4–18.1)	(7.5–11.6)	(4.4–5.2)	(6.8–7.8)	(10.1–15.9)	(20.5–26.6)
Estonia	F	22.7	57.6	12.8	5.9	2.1	4.3	4.8	18.4
		(19.8–25.9)	(52.6–62.4)	(8.6–18.5)	(4.4–7.8)	(1.9–2.4)	(3.9–4.6)	(3.4–6.8)	(15.7–21.5)
Hungary	F	28.1	40.3	21.9	3.4	3.0	7.1	7.2	12.9
		(24.7–31.7)	(34.7–46.1)	(16.3–28.6)	(2.3–5.1)	(2.5–3.5)	(6.4–7.9)	(4.5–11.2)	(10.5–15.6)
Lithuania	F	19.1	60.0	13.7	9.4	3.7	6.3	10.9	21.1
		(16.1–22.4)	(54.3–65.5)	(8.3–21.7)	(7.4–11.9)	(3.1–4.2)	(5.8–6.8)	(8.2–14.2)	(18.1–24.5)
Poland	F	25.6	49.9	26.1	6.2	2.2	4.9	5.8	16.1
		(22.2–29.2)	(44.2–55.6)	(19.6–33.8)	(4.6–8.5)	(1.9–2.6)	(4.4–5.3)	(4.0–8.4)	(13.3–19.4)
Slovenia	F	27.9	51.0	24.4	9.5	2.0	2.5	3.1	19.9
		(23.9–32.2)	(45.0–57.1)	(17.5–32.8)	(7.1–12.4)	(1.6–2.3)	(2.2–2.8)	(1.7–5.5)	(16.5–23.9)
Israel	F	20.6	33.6	35.4	4.2	2.0	3.5	9.9	18.4
		(17.8–23.6)	(27.9–39.9)	(28.4–43.1)	(3.1–5.8)	(1.7–2.4)	(3.0–4.0)	(6.9–14.0)	(15.7–21.4)
Portugal	F	21.3	41.3	14.8	14.6	2.0	3.0	5.1	12.4
		(17.1–26.2)	(33.7–49.4)	(8.7–24.1)	(11.3–18.7)	(1.6–2.3)	(2.4–3.5)	(2.9–8.8)	(9.3–16.4)
Spain	F	30.8	46.0	20.3	16.7	1.2	3.3	7.6	16.6
		(27.4–34.5)	(40.9–51.1)	(15.4–26.4)	(14.1–19.7)	(1.1–1.3)	(3.0–3.5)	(5.5–10.4)	(13.9–19.7)

Note: Sample consists of individuals living in Greece aged between 20 and 64. CIs, confidence intervals.

**Table 9:** Social determinants of health in European countries (95% CIs)

Country		Any ergonomic hazards	Any material hazards	Often/always conflict growing up	Often/always hardship growing up	Provide unpaid care	>10 h of unpaid care/week
ESS pooled	M	63.9 (63.0–64.9)	59.1 (58.1–60.0)	10.2 (9.6–10.8)	13.4 (12.7–14.1)	30.3 (29.5–31.2)	18.0 (16.6–19.5)
	F	49.6 (48.7–50.5)	36.5 (36.5–37.4)	14.1 (13.5–14.8)	16.0 (15.3–16.6)	36.3 (35.5–37.2)	26.5 (25.2–28.0)
Greece	M	49.1 (42.8–55.4)	51.5 (45.1–57.8)	8.0 (5.1–12.4)	23.9 (19.3–29.3)	13.1 (9.5–17.7)	34.2 (22.4–48.4)
	F	36.7 (30.9–42.9)	30.4 (25.0–36.5)	8.7 (5.7–12.9)	22.7 (18.2–27.9)	20.0 (15.9–24.9)	51.5 (37.5–65.2)
Denmark	M	71.8 (67.5–75.6)	64.5 (60.1–68.7)	14.6 (11.7–18.2)	12.6 (9.7–16.2)	41.6 (37.2–46.1)	17.2 (11.4–24.9)
	F	65.6 (61.1–69.9)	54.0 (49.3–58.7)	21.0 (17.4–25.3)	16.7 (13.5–20.6)	46.7 (42.1–51.4)	15.0 (9.9–22.1)
Finland	M	83.6 (80.7–86.2)	77.7 (74.4–80.7)	8.6 (6.8–10.9)	10.5 (8.5–13.0)	40.9 (37.2–44.6)	9.1 (6.2–13.3)
	F	79.0 (75.7–82.0)	56.7 (52.8–60.5)	16.6 (13.9–19.7)	16.6 (13.9–19.8)	47.2 (43.4–51.0)	9.8 (6.8–13.8)
Norway	M	63.8 (59.6–67.8)	62.4 (58.1–66.4)	5.4 (3.7–7.8)	5.4 (3.7–7.8)	35.2 (31.1–39.4)	6.0 (3.2–11.1)
	F	56.9 (52.0–61.6)	41.9 (37.3–46.7)	12.3 (9.2–16.2)	9.6 (7.1–13.0)	46.0 (41.3–50.8)	11.2 (7.2–17.0)
Sweden	M	73.5 (69.7–77.0)	70.0 (66.1–73.7)	11.7 (9.2–14.7)	9.7 (7.3–12.8)	39.0 (35.2–43.0)	8.4 (5.4–12.8)
	F	68.8 (64.9–72.4)	51.0 (46.9–55.2)	17.6 (14.7–21.1)	14.2 (11.2–17.7)	40.0 (35.9–44.1)	15.0 (10.9–20.4)
Austria	M	57.5 (53.1–61.8)	49.7 (45.5–53.9)	8.7 (6.5–11.6)	10.9 (8.5–13.9)	17.2 (14.2–20.7)	21.3 (14.3–30.5)
	F	38.8 (34.9–42.9)	25.2 (21.8–28.9)	14.2 (11.5–17.4)	12.3 (9.8–15.2)	26.1 (22.7–29.8)	35.4 (27.4–44.4)
Belgium	M	67.5 (63.6–71.1)	64.0 (60.1–67.7)	12.8 (10.4–15.8)	12.7 (10.2–15.8)	36.3 (32.7–40.2)	13.6 (9.6–18.8)
	F	52.6 (48.5–56.6)	34.5 (30.7–38.5)	18.0 (15.2–21.3)	13.7 (11.1–16.8)	40.2 (35.6–44.1)	17.8 (13.3–23.5)
France	M	74.5 (70.4–78.3)	70.3 (65.9–74.3)	12.4 (9.7–15.7)	13.9 (10.9–17.7)	36.4 (32.0–41.1)	16.1 (9.9–25.0)
	F	60.4 (55.9–64.7)	39.9 (35.6–44.4)	21.4 (17.9–25.3)	21.5 (17.9–25.5)	39.9 (35.6–44.4)	18.3 (13.5–24.5)
Germany	M	73.6 (70.6–76.4)	69.0 (65.8–72.0)	13.6 (11.4–16.3)	12.3 (10.1–14.8)	32.5 (29.4–35.7)	15.9 (11.9–20.8)
	F	57.5 (54.1–60.8)	41.0 (37.7–44.3)	19.3 (16.7–22.1)	12.5 (10.4–15.0)	37.8 (34.7–41.1)	17.3 (13.6–21.8)
Ireland	M	48.6 (44.5–52.8)	43.6 (39.4–47.8)	7.4 (5.6–9.9)	16.8 (14.1–20.0)	20.1 (17.1–23.5)	32.1 (23.8–41.8)
	F	31.8 (28.1–35.8)	27.9 (24.3–31.8)	9.1 (7.2–11.4)	15.5 (13.2–18.2)	30.7 (27.4–34.2)	43.0 (36.6–49.6)
Netherlands	M	62.6 (58.1–67.0)	57.1 (52.3–61.7)	10.5 (8.1–13.4)	12.2 (9.5–15.4)	32.5 (28.4–36.9)	21.0 (14.0–30.2)
	F	48.7 (44.7–52.8)	31.7 (28.0–35.6)	15.5 (12.9–18.4)	10.3 (8.1–13.0)	38.8 (35.1–42.6)	15.9 (11.6–21.4)
Switzerland	M	57.2 (53.0–61.3)	53.4 (49.2–57.6)	11.1 (8.7–14.1)	11.1 (8.7–14.2)	33.3 (29.5–37.4)	10.9 (6.7–17.4)
	F	43.4 (39.2–47.7)	30.8 (27.0–35.0)	17.7 (14.7–21.2)	12.7 (10.2–15.8)	41.2 (37.2–45.3)	16.4 (11.7–22.4)
UK	M	64.2 (59.8–68.4)	60.6 (56.2–64.8)	14.2 (11.4–17.5)	14.9 (12.3–18.0)	29.1 (25.3–33.2)	30.0 (22.8–38.3)
	F	42.8 (39.0–46.7)	33.0 (29.5–36.7)	17.9 (15.2–21.0)	20.4 (17.6–23.5)	31.1 (27.8–34.5)	26.9 (21.8–32.8)
Czech Republic	M	46.8 (42.5–51.1)	45.4 (41.1–49.6)	7.3 (5.5–9.8)	12.2 (9.6–15.3)	34.0 (30.2–38.0)	14.7 (10.4–20.5)
	F	30.0 (26.4–33.9)	20.4 (17.3–23.9)	7.3 (5.6–9.5)	13.6 (11.2–16.4)	39.1 (35.6–42.7)	35.1 (29.6–40.9)
Estonia	M	71.7 (67.9–75.2)	63.5 (59.5–67.3)	10.2 (7.9–13.0)	19.6 (16.6–23.0)	28.6 (25.1–32.3)	26.5 (19.8–34.5)
	F	51.4 (47.7–55.1)	42.4 (38.8–46.0)	17.1 (14.5–20.1)	20.4 (17.6–23.4)	36.5 (33.1–40.0)	42.5 (36.3–49.0)
Hungary	M	55.6 (51.0–60.0)	43.8 (39.3–48.3)	11.7 (9.1–15.0)	18.6 (15.3–22.5)	7.0 (5.1–9.5)	31.0 (21.3–42.6)
	F	35.2 (31.4–39.2)	25.3 (21.8–29.0)	10.8 (8.5–13.7)	19.6 (26.5–23.2)	10.9 (8.7–13.5)	35.5 (25.6–46.8)
Lithuania	M	60.1 (55.3–64.7)	44.9 (40.1–49.8)	11.3 (8.6–14.8)	15.8 (12.6–19.7)	14.5 (11.6–18.0)	30.9 (20.4–43.8)
	F	36.3 (32.4–40.3)	30.1 (26.3–34.2)	11.0 (8.9–13.5)	18.9 (16.1–22.1)	25.1 (21.9–28.6)	44.4 (35.4–53.7)
Poland	M	71.5 (67.5–75.2)	75.0 (71.1–78.5)	8.0 (6.1–10.6)	14.0 (11.3–17.1)	34.3 (30.4–38.4)	17.0 (12.0–23.5)
	F	56.0 (51.8–60.1)	45.3 (41.2–49.5)	7.9 (6.0–10.4)	15.8 (13.0–19.1)	40.1 (36.2–44.1)	35.6 (29.5–42.2)
Slovenia	M	62.7 (57.3–67.7)	64.8 (59.5–69.8)	4.9 (3.2–7.5)	9.4 (6.8–12.8)	33.3 (28.6–38.3)	11.6 (7.5–17.3)
	F	55.7 (50.6–60.7)	41.6 (36.7–46.8)	15.3 (12.2–19.0)	18.8 (15.6–22.5)	39.1 (34.7–43.6)	20.1 (15.0–26.3)
Israel	M	45.0 (41.0–49.1)	43.4 (39.5–47.5)	11.8 (9.2–15.0)	15.8 (12.9–19.1)	37.1 (33.2–41.1)	16.7 (12.1–22.6)
	F	32.2 (28.6–36.0)	24.2 (21.0–27.7)	11.7 (9.5–14.3)	22.2 (19.2–25.5)	40.2 (36.7–43.8)	32.8 (27.3–38.9)
Portugal	M	69.6 (63.7–74.9)	62.4 (56.4–68.1)	8.2 (5.1–12.8)	20.0 (15.3–25.7)	36.1 (30.4–42.1)	32.0 (22.8–42.9)
	F	61.3 (55.3–66.9)	41.7 (36.0–47.5)	11.5 (8.1–16.1)	16.7 (13.2–20.9)	35.1 (29.9–40.8)	40.4 (31.6–49.8)
Spain	M	71.0 (67.2–74.5)	65.1 (61.2–68.8)	5.1 (3.7–7.0)	11.5 (9.4–14.1)	24.5 (21.5–27.8)	30.7 (24.2–38.2)
	F	57.3 (53.5–61.3)	40.9 (37.0–45.0)	7.0 (5.2–9.3)	11.2 (9.1–13.8)	36.7 (33.2–40.3)	40.9 (34.8–47.2)

Note: Sample consists of individuals living in Greece aged between 20 and 64. CIs, confidence intervals.

### Non-communicable diseases

Overall, respondents in both surveys were asked about 12 different NCDs. The answer categories were either 0 = did not experience the NCD in the past 12 months or 1 = experienced it. The prevalence of each of the 12 diseases for Greece and the 21 ESS countries are given in table 5—separately for men and women. Apart from the prevalence of each disease, we show the prevalence of reporting at least one or two of these diseases. Moreover, we show the occurrence of cancer—either currently or previously.

Table 5 suggests that the prevalence of NCDs is lower than the overall ESS average for both Greek men and women. The summarized disease index for Greece is lower than that in most of the other countries, whilst the values are similar to those in the other Southern European countries, namely Portugal and Spain.

### Self-reported health measures

Table 6 shows the prevalence of reporting poor or very poor general health. In contrast to the above-mentioned results in NCDs, we see a slightly higher prevalence of poor health among Greek women, while the prevalence of poor health among Greek men is lower than the ESS pooled value. Another measure of general health is whether or not one feels hampered in one’s daily life. Again, Greek prevalence is considerably below the ESS average. Only 8.6% of men and 13.7% of women in the Greek sample feel hampered in their daily life.

The prevalence of self-reported depressive symptoms is exceptionally high for Greece compared with the ESS average (table 6). While the ESS average for men is 9.5%, 26.2% of Greek men reported depressive symptoms. There is, moreover, quite a sizeable gap between men and women in the Greek sample: prevalence of depression among women is 11 percentage points higher than among men (37.1%).

Lastly, the prevalence of overweight or obesity is presented in table 6. Greek respondents are in line with the ESS average. In general, the tendency in most countries is that men are more often overweight than women.

### Healthcare use

As previously mentioned, there were significant changes in the provision of healthcare in Greece during the crisis. Table 7 presents the different measures for healthcare use: whether needs were met or not, the reasons why needs were not met, whether the respondent went to see a general practitioner (GP) or a specialist or made use of alternative treatments.

The measure of unmet need captures the subjective perception of not receiving appropriate treatment during the last 12 months. Women are more likely to report unmet needs than men. In the Greek sample, this difference is quite significant and stands at 12 percentage points. We also see that compared with the ESS overall average, the Greek respondents more often reported that their needs were not met as a result of being put on a waiting list or because there was no appointment available.

Interestingly, we find a very low prevalence of GP consultation among both Greek men and women. The Greek average lies roughly 50 percentage points below the ESS average. No other country reports such a low GP consultation prevalence. By contrast, specialist use is fairly widespread: Greek respondents report visiting a specialist more than the respondents in the pooled ESS sample do, with women visiting specialists more often than men in the Greek sample. Lastly, the use of alternative treatments does not seem to be very prevalent in Greece: only 8.1% of men and 11.9% of women make use of alternative treatments. These values are roughly 22 and 30 percentage points, respectively, below the ESS average.

### Risk behaviour

In general, the Greek sample reported better general health—apart from depressive symptoms—than the average population in the ESS. However, we cannot attribute it to the healthy behaviour of the Greek population. On the contrary, health risk behaviours are common (tables 8a and b). Both women and men in Greece, on average, smoke and drink more than the average ESS population. Moreover, while we see large differences in the smoking habits between men and women in most of the 21 ESS countries, there is almost no difference between the two groups in the MIGHEAL sample. Moreover, among those who smoke in the Greek sample, 50.5% of men and 46.4% of women reported smoking more than 20 cigarettes a day. In the pooled ESS, only 38% of men and 22% of women reported smoking more than a package a day.

In addition to heavy smoking habits, 42% of men in the MIGHEAL sample reported that they binge drink at least once a week. That is roughly 18 percentage points higher than the ESS average. Even though Greek women reported a lower prevalence of binge drinking (24.3%), they are still 12 percentage points above the pooled ESS average. Lastly, the prevalence of physical activity in the Greek samples resembles the average physical activity in the ESS countries.

### Social determinants of health

Individual health is determined not only by risky health behaviour but also by social circumstances. Table 9 shows the prevalence of ergonomic and material hazards, negative experiences while growing up, as well as respondents’ unpaid care responsibilities.

According to the reported prevalence rates in table 9, Greek respondents do not or did not experience more ergonomic or material hazards than the ESS average. Their average hazards are lower than the ESS value. They also, on average, have experienced less conflict at home while growing up, although they experienced more economic hardship while growing up compared with the pooled ESS average.

Lastly, while we find a lower prevalence of providing unpaid care among both Greek men and women than the ESS average, those who have caring responsibilities spend on average more time on them than the respondents from the ESS countries (overall average). For example, 51% of Greek women provide more than 10 hours of unpaid care per week, in contrast to 26.5% of women in the pooled ESS sample.

### The ranking of Greece among European countries concerning health and health determinants

Table 10 sums up the findings presented above by ranking the Greek values in relation to the 21 ESS countries. Most notably, Greece shows the highest prevalence of self-reported depressive symptoms out of the 22 countries. It is also among the highest ranking countries in terms of smoking and drinking behaviour, as well as experiencing economic hardship at home while growing up and the number of hours of unpaid care provided. Greek respondents visit GPs the least often and make less of use of alternative treatments.

**Table 10:** Greece—rank within Europe

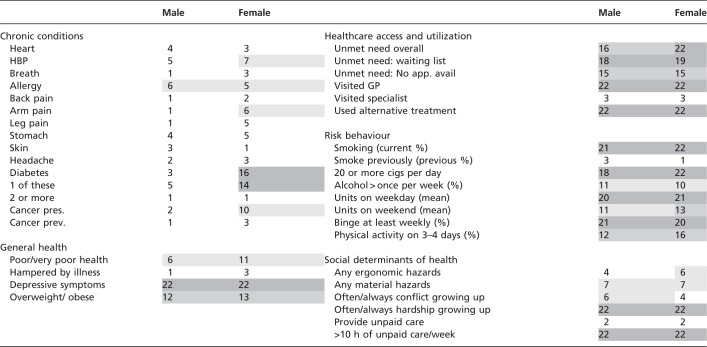

Note: Rank within tables presenting prevalence rates. Darker colour indicates lower rank.

### Regression results according to citizenship

In this section, we analyze the odds of reporting these health conditions and of showing risky behaviour and social determinants of health, depending on citizenship. In other words, health status and behaviour might differ according to a person’s cultural background—in this case depending on whether an individual has Greek, Albanian or other country’s citizenship. Table 11 summarizes our findings. Values lower than 1 indicate lower odds of showing a particular indicator compared with Greek citizens, while higher values indicate higher odds. Only the estimates for predicted mean units per weekday/weekend day are not based on odds ratios.

**Table 11:** Odds ratios (95% CIs) according to citizenship

	Greek	Albanian	Other		Greek	Albanian	Other
Chronic conditions				Healthcare access and utilization			
Heart	1	**0.33 (0.10–1.15)**	1.38 (0.41–4.57)	Unmet need overall	1	1.02 (0.67–1.56)	1.25 (0.61–2.58)
HBP	1	**0.35 (0.14–0.87)**	**0.27 (0.09–0.80)**	Unmet need: waiting list	1	0.92 (0.50–1.69)	**0.38 (0.16–0.89)**
Breath	1	0.43 (0.13–1.47)	0.92 (0.35–2.37)	Unmet need: No app. avail	1	1.10 (0.57–2.12)	1.07 (0.27–4.24)
Allergy	1	0.57 (0.15–1.22)	0.83 (0.34–2.03)	Visited GP	1	0.87 (0.50–1.51)	0.60 (0.23–1.54)
Back pain	1	**0.49 (0.27–0.89)**	1.22 (0.49–3.01)	Visited specialist	1	**0.61 (0.42–0.87)**	**0.56 (0.31–1.03)**
Arm pain	1	1.03 (0.52–2.03)	1.43 (0.49–4.21)	Used alternative treatment	1	**0.16 (0.06–0.39)**	1.53 (0.54–4.38)
Leg pain	1	0.75 (0.37–1.52)	2.01 (0.79–5.11)				
Stomach	1	**0.39 (0.19–0.81)**	**0.29 (0.11–0.76)**	Risk behaviour			
Skin	1	0.34 (0.04–2.54)	0.43 (0.12–1.60)	Smoking (current %)	1	**0.49 (0.34–0.71)**	0.72 (0.35–1.47)
Headache	1	0.56 (0.25–1.23)	0.58 (0.15–2.33)	Smoke previously (previous %)	1	0.96 (0.54–1.68)	1.09 (0.30–3.88)
Diabetes	1	0.34 (0.09–1.25)	1.87 (0.66–5.32)	20 or more cigs per day	1	1.21 (0.45–1.05)	**0.29 (0.09–0.86)**
1 of these	1	**0.51 (0.33–0.79)**	1.44 (0.70–2.95)	Alcohol > once per week (%)	1	**0.69 (0.45–1.05)**	**0.16 (0.05–0.54)**
2 or more	1	**0.53 (0.32–0.90)**	0.70 (0.33–1.50)	Predicted mean units on a weekday	**5.86 (3.81–8.36)**	**6.94 (4.40–9.05)**	**2.43 (–0.25–4.40)**
Cancer presently	1	0.19 (0.02–1.62)	0.19 (0.02–1.61)	Predicted mean units on a weekend-day	**6.46 (5.12–8.07)**	**6.83 (5.19–8.14)**	**3.38 (1.50–4.45)**
Cancer previously	1	0.44 (0.12–1.62)	**0.33 (0.09–1.23)**	Binge at least weekly (%)	1	0.91 (0.59–1.40)	0.62 (0.18–2.08)
				Physical activity on 3–4 days (%)	1	0.70 (0.43–1.13)	0.82 (0.33–2.03)
General health							
Poor/very poor health	1	0.54 (0.18–1.26)	**0.25 (0.08–0.84)**	Social determinants of health			
Hampered by illness	1	**0.45 (0.22–0.92)**	1.07 (0.41–2.80)	Any ergonomic hazards	1	**4.95 (3.33–7.36)**	**4.09 (2.00–8.38)**
Depressive symptoms	1	0.97 (0.65–1.45)	0.59 (0.27–1.30)	Any material hazards	1	**3.77 (2.59–5.48)**	**2.66 (1.37–5.16)**
Overweight/ obese	1	0.92 (0.63–1.34)	0.73 (0.34–1.53)	Often/ always conflict growing up	1	**0.36 (0.16–0.80)**	0.83 (0.30–2.34)
				Often/ always hardship growing up	1	**2.92 (1.97–4.34)**	**1.95 (1.01–3.78)**
				Provide unpaid care	1	0.80 (0.48–1.33)	0.40 (0.14–1.19)
				>10 h of unpaid care/week	1	1.94 (0.63–5.93)	**0.13 (0.03–0.64)**

Note: Sample consists of individuals living in Greece aged between 20 and 64. Bold faced numbers indicate significant associations. CIs, confidence intervals.

There are not many significant differences between the three different citizenships (bold estimates): Albanians are significantly less likely to report chronic conditions, i.e. having at least one or two. There is no difference between native Greeks and citizens of other countries. Yet they are significantly less likely to report poorer health than native Greeks.

Moreover, both migrant groups are less likely to visit a specialist or use alternative treatments. They also have lower odds of smoking and drinking alcohol more than once a week. However, Albanians drink more units of alcohol both on weekdays and weekend days, whereas other countries’ citizens drink fewer units.

The biggest differences in the odds between the three citizenship groups can be found in the social determinants of health: both migrant groups (Albanians and other country migrants) have significantly higher odds of reporting both ergonomic and material hazards. They also have higher odds of having experienced economic hardship while growing up, even though the Greek values were already the highest in the European comparison (see above).

### Self-reported depressive symptoms in MIGHEAL and ESS

The MIGHEAL data show a high prevalence of depression among native women. A comparison of depressive symptoms with ESS pooled results, as well as with individual ESS countries, indicates that the native female population is at high risk of depression (see table 6).

Rates for depressive symptoms in Greece are at alarming levels: 37% for women and 26% for men. These rates are much higher than those in any other country in the ESS and greatly exceed the pooled ESS levels of 18.8% and 10.2%, respectively.

The countries exhibiting the highest levels in the ESS survey (albeit substantially below Greek levels) are the Czech Republic (22.5%) and Portugal (24.7%), both reported for women. Recent analysis of ESS data over time (2006–12) has indicated an increasing trend in self-reported depression in Cyprus and Spain and a substantial rise in depression rates for the unemployed in comparison to Hungary.^20^ Those who are precariously employed or inactive in the labour market in Europe are at higher risk of depression.^21^^,^^22^ Similarly, first-generation EU and non-EU migrants are at higher risk of depression compared with second-generation and native populations in Europe.^23^ But as Leveque and van Rossem have shown, barriers to socio-economic integration and perceived discrimination rather than ethnicity and minority status are the drivers of depression for migrants.^24^

Due to the high prevalence of self-reported depressive symptoms in Greece, the original items in the depression scale were broken down individually. The graphs below present each of the eight items, with the original coding from ‘none of the time’ to ‘all of the time’ (figure 2). These items have not been age-standardized, but full MIGHEAL and ESS pooled age distributions are quite similar.

**Figure 1:**
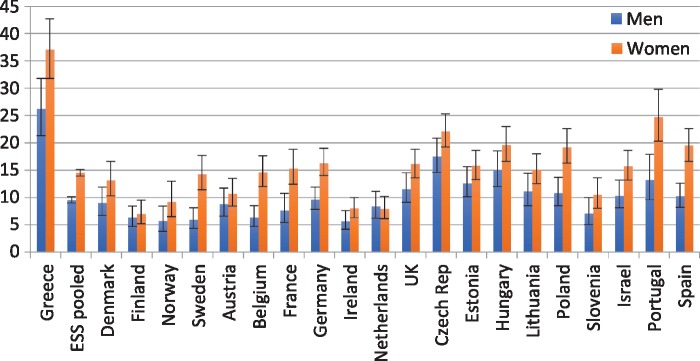
Self-reported depressive symptoms in Greece (MIGHEAL data) and European countries (ESS data) (95% confidence intervals)

**Figure 2:**
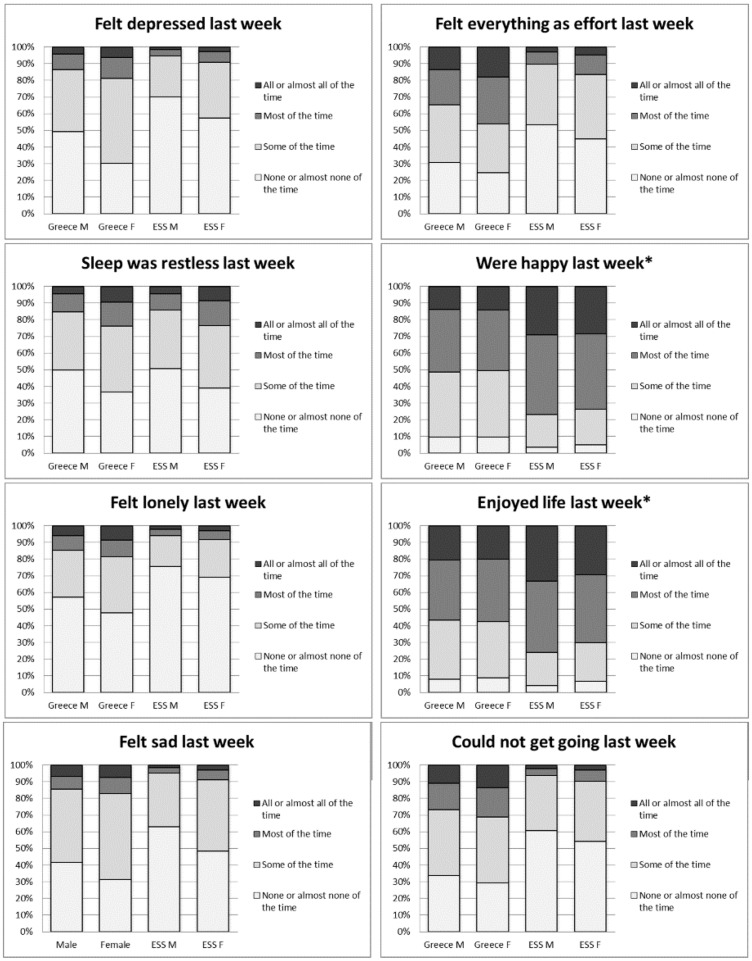
Items in depression scale in Greece (MIGHEAL data) and European countries (ESS data). *indicates reversed values

Major differences between the ESS and MIGHEAL data appear in the following items of the depression scale: ‘feeling depressed’, ‘high effort’, ‘being happy’, ‘enjoying life’, ‘feeling sad’ and ‘could not get going’, where Greece fares worse. Fifty per cent of Greeks reported ‘not feeling happy’ most or all of the time, compared with around 25% in the ESS. Only in the item ‘feeling lonely’ did Greece compare favourably with the ESS. Women responded negatively on almost all items.

## Discussion

The MIGHEAL survey was conducted among migrants and non-migrants in Greece to investigate inequalities in self-reported health, healthcare use and unmet healthcare needs between the migrant and the native population.

The survey provides evidence at the national level regarding social inequalities in health and healthcare use; however, no significant differences were found between migrants and natives with respect to unmet need. The European comparison, on the other hand, revealed that Greeks report rather low levels of poor or very/poor self-reported health as well as lower levels of NCDs. In contrast, they have the highest prevalence of self-reported depressive symptoms across Europe.

With respect to self-reported health, the findings reveal that women, regardless of their citizenship status, are more likely to report very poor or poor health than men. They also tend to report more NCDs than men (see table 5). There seems to be a general tendency among women to report health issues more often than men. This gender gap in self-reported health measures is in line with previous research.^25^ An earlier study based on the ESS shows that women in Europe tend to report poorer health status than men, with higher percentages in South-East European countries.^23^

Differences in self-reported health were only found among citizens of other countries. However, these findings indicate that they have lower odds of reporting poor or very poor health. These findings are in line with the so-called ‘healthy immigrant paradox’.^26^

As for healthcare use, the MIGHEAL study shows that the rate of GP visits is low for all groups in the Greek sample compared with the pooled ESS average, while rates for no medical visits are high compared with ESS findings. Socio-economic differences in healthcare services utilization have been widely reported in European countries.^27^ Moreover, people from the lower socio-economic strata tend to be more intensive users of GPs, while members of the upper socio-economic strata report significantly more visits to specialists. Hence, the presented findings in the Greek sample reveal a different pattern in healthcare use compared with Europe: the native-born population tends to visit specialists rather than going to a GP. The pattern of doctor visits is completely reversed in Greece compared with that of ESS countries. Yet we also find a reversed pattern for the migrant population: migrants show significantly lower odds of visiting a specialist than Greeks. According to the literature, various possible reasons for disparities in healthcare services have been suggested, including systematic differences in the interpretation of symptoms and the perceptions of the need for healthcare by socio-economic position and ethnicity.^28^

In contrast to previous studies based on ESS data indicating that migrants in European countries have slightly higher odds of reporting unmet need,^29^ our analysis showed that there is no significant difference between migrant and non-migrant groups in Greece, even though migrants report slightly higher percentages. Our findings also reveal a gender gap, with women reporting the highest percentages of unmet need in healthcare, with waiting lists being cited as the most common reason. These results are in line with previous studies that show the association of unmet need and financial strain^29^ and report higher rates of unmet need among individuals with lower income status.^30^

A comparison with findings from two recent Greek surveys on health spending and unmet needs,^^31^^ which focused on the general population (no distinction made between migrant and non-migrant respondents), suggests that access to healthcare has become increasingly difficult for the population: over half of the households reported delaying their visit to the doctor and their treatment. This appears to be the result of the ongoing crisis and the austerity policies pursued since 2010.^32^ It is interesting to note that despite the significant fall in household incomes, private health expenditure has been increasing in the wake of continuous drastic public spending cuts in the health sector. Greece reports the highest rate of private spending on health services in the EU. Since 2009, the private share of health spending increased by around 4 percentage points, representing 31% of total health spending in 2014. However, in monetary terms, due to shrinking incomes, spending on health services was reduced by 14% between 2010 and 2015. A reduction which is, nonetheless, the smallest recorded among the 12 categories of spending.^33^ Additionally, a recent survey on the impact of the crisis on the health of the Greek population by the Greek Institute of Social and Preventive Medicine^34^ supports the established view that health and mental health are deteriorating, in particular among the lower socio-economic strata. The Institute also reports a rise in unmet health needs, due to costs and cuts in the National Health System. The problem is particularly acute among the unemployed, pensioners and those with a lower socio-economic position. According to this study, 65% of respondents reported that they had to pay out of pocket for the medical care they received during the past 12 months. Those near or under the poverty line had to pay a larger fraction of their low income to cover medicine and healthcare.

Levels of unmet need for healthcare were also found to be alarmingly high in Greece compared with ESS countries, suggesting that the crisis and subsequent austerity policies may have severely impacted the provision of healthcare services and access to healthcare for broad sections of the population, whether native or migrant.

The MIGHEAL survey has documented a high prevalence of depressive symptoms in the Greek population. These results are in line with previous evidence.^35^^,^^36^ Compared with levels recorded in the ESS, exceptionally high levels of depressive symptoms were found particularly among native females, thus suggesting that Greek women are at high risk of suffering from depression. A possible explanation is that women in Greek society are particularly burdened by their role as informal care providers for children and the elderly, a role that compromises their working potential and independence from the family. Whether in paid employment or not, it is women who are mainly responsible for domestic work and care work.^37^ As has been argued, in times of crisis, a drastic fall in household disposable income may make women more vulnerable, thus widening the gender gap in depression.^23^

## Conclusion

The MIGHEAL study has been able to document a complex pattern of inequalities among and between population sub-groups, lending support to the healthy immigrant theory. However, the potential of the MIGHEAL study goes far beyond within-country comparisons. The survey constitutes the most recent and comprehensive overview of self-reported conditions and their determinants in Greece within the European context, which can and should be further elaborated in future comparative studies. The initial results presented here provide evidence on health inequalities between men and women and between migrant groups, thus fleshing out the pan-European documentation provided by ESS. Moreover, our findings are in line with prior research that has associated the prolonged crisis in Greece with adverse health outcomes.
